# Postpartum haemorrhage occurring in UK midwifery units: A national population-based case-control study to investigate incidence, risk factors and outcomes

**DOI:** 10.1371/journal.pone.0291795

**Published:** 2023-10-05

**Authors:** Madeline Elkington, Jennifer J. Kurinczuk, Dharmintra Pasupathy, Rachel Plachcinski, Jane Rogers, Catherine Williams, Rachel Rowe, on behalf of the UKMidSS Steering Group

**Affiliations:** 1 NIHR Policy Research Unit in Maternal and Neonatal Health and Care, National Perinatal Epidemiology Unit, Nuffield Department of Population Health, University of Oxford, Oxford, United Kingdom; 2 Reproduction and Perinatal Centre, Faculty of Medicine and Health, University of Sydney, Sydney, Australia; 3 Independent Parent, Patient and Public Involvement Consultant, Dewsbury, United Kingdom; 4 Consultant Midwife, Formerly at University Hospitals Southampton, Southampton, United Kingdom; 5 Independent Parent, Patient and Public Involvement Consultant, Henley on Thames, United Kingdom; Kasr Alainy Medical School, Cairo University, EGYPT

## Abstract

**Objectives:**

To estimate the incidence of, and investigate risk factors for, postpartum haemorrhage (PPH) requiring transfer to obstetric care following birth in midwifery units (MU) in the UK; to describe outcomes for women who experience PPH requiring transfer to obstetric care.

**Methods:**

We conducted a national population-based case-control study in all MUs in the UK using the UK Midwifery Study System (UKMidSS). Between September 2019 and February 2020, 1501 women with PPH requiring transfer to obstetric care following birth in an MU, and 1475 control women were identified. We used multivariable logistic regression, generating adjusted odds ratios (aORs) and 95% confidence intervals (CIs) to investigate risk factors for PPH requiring transfer to obstetric care.

**Results:**

The incidence of PPH requiring transfer to obstetric care following birth in an MU was 3.7% (95% CI 3.6%-3.9%). Factors independently associated with PPH requiring transfer to obstetric care were smoking during pregnancy (aOR = 0.73; 95% CI 0.56–0.94), nulliparity (aOR = 1.96; 95% CI 1.66–2.30), previous PPH (aOR = 2.67; 95% CI 1.67–4.25), complications in a previous pregnancy other than PPH (aOR = 2.40; 95% CI 1.25–4.60), gestational age ≥41 weeks (aOR = 1.36; 95% CI 1.10–1.69), instrumental birth (aOR = 2.69; 95% CI 1.53–4.72), third stage of labour ≥60 minutes (aOR = 5.56; 95% CI 3.93–7.88), perineal trauma (aOR = 4.67; 95% CI 3.16–6.90), and birthweight 3500-3999g (aOR = 1.71; 95% CI 1.42–2.07) or ≥4000g (aOR = 2.31; 95% CI 1.78–3.00). One in ten (10.6%) cases received a blood transfusion and one in five (21.0%) were admitted to higher level care.

**Conclusions:**

The risk factors identified in this study align with those identified in previous research and with current guidelines for women planning birth in an MU in the UK. Maternal outcomes after PPH were broadly reassuring and indicative of appropriate management. NHS organisations should ensure that robust guidelines are in place to support management of PPH in MUs.

## Introduction

Childbirth in the UK is generally safe for women and their babies, and complications following birth are relatively rare, prticularly for women who are healthy with straightforward pregnancies. Most women who give birth in the UK do so in a consultant-led obstetric unit (OU), however, around 13% of births occur in midwifery units (MUs) [1]. Since the early 1990s, support for women’s choices, specifically around place of birth, has been a central focus of UK maternity care policy [2]. Since 2014, national guidance has explicitly recommended that women at low risk of complications should be able to choose between birth at home, in an MU or in an OU [3]. In the UK, MUs may be either located on the same site as an OU, referred to as ‘alongside’ midwifery units (AMUs), or in a separate location from an OU, freestanding midwifery units (FMUs). Care in MUs is provided by midwives, and transfer to an OU is required for obstetric or medical care [3]. In an AMU, this may involve moving to another ward or floor in the same hospital, or, in some cases, the woman may remain in the AMU while receiving care from an obstetrician. Transfer from an FMU typically takes place by ambulance. The current national guideline suggests that planning birth in an MU is particularly suitable for women who are healthy with straightforward pregnancies because it is associated with a lower risk of intervention and no increased risk of adverse outcome for mothers or babies [3].

Many of the risk factors for postpartum haemorrhage (PPH), excessive bleeding after childbirth, including previous caesarean section [4], multiple gestation [5] and hypertension [6, 7] are less common in women who plan birth in an MU because women with these risk factors are generally advised to plan birth in an OU [3]. Admission criteria vary between MUs, however. A survey of admission criteria for MUs found that most guidelines (86%) listed at least one criterion that was considered ‘more inclusive’ than the national guidelines, that is, admitting women who have one or more risk factor identified in the NICE guidelines [8]. Other research using data from UK MUs confirms that women who might be considered to be at a higher risk of complications, including those with a pre-existing medical risk factor or pregnancy complication, are admitted to MUs, albeit in relatively small numbers [9, 10]. Women at low risk of complications who plan birth in MUs are less likely to develop complications during labour and birth, including PPH, compared with women at low risk of complications who plan birth in an OU [11]. However, women admitted to MUs may still experience a PPH; some after transfer to obstetric care and some following birth in an MU. In an MU setting, there may be a delay in access to medical treatments for PPH such as blood products or an operating theatre, particularly in FMUs, where a transfer for medical care necessarily involves travel to a different location. To ensure births in an MU are as safe as possible, recognition of women who may be at increased risk of a PPH and the prompt diagnosis and management of a PPH is critical.

There is currently limited evidence about how often PPH occurs in MUs, the risk factors for PPH among women who give birth in MUs or outcomes for women who experience PPH in MUs. Such evidence would help inform decision-making for women considering or planning birth in an MU and the health professionals providing their care.

This study aimed to (a) estimate the incidence of PPH requiring transfer to obstetric care following birth in an MU in the UK, (b) investigate the risk factors for PPH requiring transfer to obstetric care among women who give birth in MUs in the UK, and (c) identify risk factors for admission to higher level care or blood transfusion, referred to below as ‘enhanced treatment or care’.

## Methods

### Study design

We carried out a national, population-based, case-control study.

### Cases and controls

Cases were identified as all women who gave birth in an MU in the UK between 1 September 2019 and 29 February 2020, who experienced a PPH requiring transfer to obstetric care, where the primary or secondary reason for transfer was PPH. One control per case was identified as the woman who did not meet the case definition who gave birth in the same MU immediately before each case.

## Data collection

We collected anonymised information from MUs using the UK Midwifery Study System (UKMidSS), a national research infrastructure involving all 127 AMUs and 79 FMUs in the UK at the time of the study. The UKMidSS infrastructure is described in detail elsewhere [12]; set up in 2015 to cover all UK AMUs, it was extended in 2019 to also involve all UK FMUs. In brief, UKMidSS comprises a network of midwife ‘reporters’ who respond to monthly email requests for data about numbers of admissions, births and ‘cases’ for UKMidSS studies. Reporters entered anonymised data for cases and controls from medical records using a secure web-based system. This study was intended to run for 12 months, however, due to the COVID-19 pandemic, and following guidance from the funder, active data collection was terminated after six months.

### Data and definitions

In the UK, at the time of data collection, blood loss volume was typically estimated visually [13], which is known to be inconsistent and inaccurate [14, 15]. We therefore used the case definition, ‘PPH requiring transfer to obstetric care’, rather than a specified blood loss volume, as a pragmatic indicator of more severe PPH. For women giving birth in FMUs, and for most women giving birth in AMUs, obstetric care for PPH would be provided in an OU, following physical transfer of the woman. In some circumstances, for some women giving birth in AMUs and experiencing PPH, an obstetrician might come into the AMU.

Maternal socio-demographic and clinical characteristics, pregnancy-related factors, intrapartum- and birth-related factors were considered as putative risk factors.

Socioeconomic status was derived from the woman’s occupation using the three-class version of the National Statistics Socio-economic Classification (NS-SEC), using the ‘simplified method’ [16]. Additional categories were created for ‘employment status unknown’, ‘employed, but occupation unrecorded/uncodable’ and ‘unemployed/student’. Where a woman was unemployed or her employment status was unknown, the partner’s occupation was used to derive socioeconomic status, if applicable. ‘Area-based deprivation quintile’ was derived using the woman’s postcode, which UKMidSS reporters entered into a bespoke website that returned a ‘score’ for the Children in Low-income Families Local Measure. This score represents the proportion of children in the area aged under 16 living in households in receipt of out of work benefits, or tax credits where their reported income is less than 60% of UK median income [17].

Women were classified as having a previous pregnancy complication if any of the following were reported: previous PPH requiring transfer or treatment, previous Caesarean section, retained placental requiring manual removal, uterine surgery other than Caesarean section. We collected information about the following pre-existing medical conditions: essential hypertension, confirmed cardiac disease, thromboembolic disorder, atypical antibodies, hyperthyroidism, diabetes, renal disease, epilepsy and ’other’ medical conditions. Information was also collected about the following current pregnancy factors: BMI at booking >35kg/m^2^, post-term pregnancy (>42 weeks), anaemia, Group B Streptococcus, antepartum haemorrhage, pre-eclampsia/pregnancy induced hypertension, gestational diabetes, malpresentation, and ‘other’. Maternal and fetal complications that, according to the national guidelines [3] may indicate the need for transfer to obstetric care, were also collected.

Data about the PPH, including estimated blood loss volume and cause and management were collected, as were neonatal (Apgar score at 5 minutes, neonatal admission to higher level care and neonatal morbidity) and maternal outcomes (admission to higher level care, maternal morbidity and blood transfusion). Among women who had a PPH requiring transfer to obstetric care, those who received a blood transfusion or were admitted to higher-level care were classified as receiving ‘enhanced treatment or care’.

### Analysis

The incidence of PPH requiring transfer to obstetric care was estimated with 95% CIs, overall and in AMUs and FMUs separately, using the number of confirmed cases as the numerator and the total number of births in MUs (and in each type of MU) during the study period as the denominator. A two-sample test of proportions was conducted to compare the incidence observed in the two types of unit.

Data entry completeness was calculated for each unit type using the number of confirmed cases as the numerator and total number of cases reported minus the number of ineligible cases as the denominator. The incidence of PPH in each unit type was calculated using the total number of cases (with complete and incomplete data) in each unit type as the numerator and the number of births in each unit type as the denominator.

The characteristics of cases and controls were described using frequencies and proportions. Univariable unconditional logistic regression was used to investigate associations between potential explanatory variables and PPH requiring transfer to obstetric care, estimating unadjusted odds ratios (ORs) with 95% CIs. Robust variance estimation was used to allow for the clustering of women within MUs.

We conducted multivariable regression analysis generating adjusted ORs (aORs) with 95% confidence intervals, using a stepwise forward regression approach. Variables with a P-value <0.1 in the univariable analysis, or where there was evidence of confounding, were considered for inclusion in the multivariable model. Variables were entered into the regression equation from distal to proximal, with sociodemographic and pre-existing risk factors being entered first, followed by pregnancy-related, intrapartum and birth-related factors. The impact of each variable as they were added was examined and assessed using the Wald test; those variables for which p<0.05 were retained in the model. We excluded ‘Administration of Syntocinon/ Syntometrine for 3rd stage management’ from the multivariable analysis, despite it being significant at the 0.05 level, because it was not possible to determine if the drug was administered in response to increased blood loss during the third stage.

Blood loss volume and maternal and neonatal outcomes among cases and controls were described using frequencies and percentages. Among cases, the causes of PPH and any ‘enhanced treatment or care’, as defined above, were tabulated using frequencies and percentages. Risk factors for ‘enhanced treatment or care’ among women who had a PPH requiring transfer were investigated using univariable logistic regression, generating ORs with 95% CIs.

We conducted two post hoc analyses to explore factors that might explain the difference in incidence of PPH in FMUs and AMUs. First we used frequencies and proportions and the Chi-square test to compare cases and controls giving birth in AMUs and FMUs using the risk factors identified as associated with PPH requiring transfer to obstetric care in the multivariable analysis. We also compared blood loss volume in cases and controls in women giving birth in different types of unit, using the same approach. Statistical significance was set at p <0.05.

### Small numbers and missing data

For privacy reasons, table cells with numbers smaller than five have been suppressed. Data completeness was high for most variables. However, data were not assumed to be missing at random and therefore a ‘Missing’ or ‘Not recorded’ category was created for all variables with ≥1% missing data. For the multivariable analysis, a complete case analysis was conducted for variables with <1% of missing data, meaning records with missing data for these variables were excluded from the analysis. For variables with ≥1% missing data, the ‘Missing’ category was included in the analysis and therefore records with missing data for these variables were retained in the analysis.

All analyses were conducted using Stata V.15.

### Sample size and power

The study was planned for a 12-month period with an anticipated incidence of PPH requiring transfer to obstetric care of 1% [18]. Based on an anticipated 107,000 births in MUs over 12 months, we estimated that the study would have 80% power at the 5% level of significance to detect ORs of 1.4 or greater and 1.7 or greater, for putative risk factors with a prevalence of 15% (e.g. gestational age >40 weeks) and 4% (e.g. current pregnancy complication), respectively. The actual number of cases and controls identified during the curtailed study period gave an estimated power of 80% at the 5% level of significance to detect ORs of 1.3 or greater and 1.6 or greater, assuming the same putative risk factors.

### Ethics

UKMidSS received approval from the National Research Ethics Service (NRES) Committee South West–Frenchay (REC ref. 15/SW/0166) in May 2015, and this study was approved as a substantial amendment to that approval (SA03) in July 2019. Because this study used anonymised data collected directly from participating units, consent from participants was not required.

## Results

### Response and incidence

All 206 MUs in the UK were invited to participate in the study and 200 units submitted at least one monthly report between September 2019 and February 2020 (97% of all UK MUs). The response to monthly report requests was 95%.

**Fig 1:**
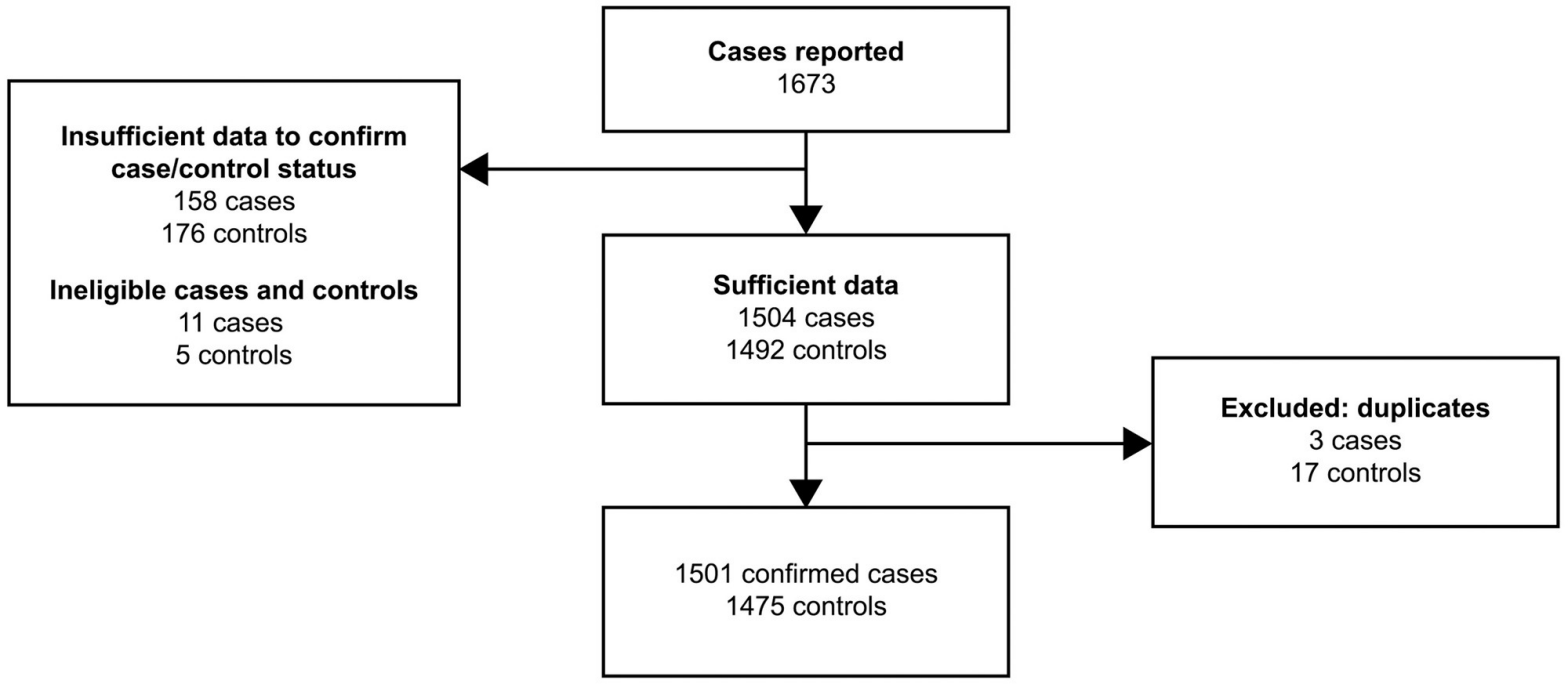
Reported and confirmed cases.

During the 6-month study period, UKMidSS midwives in participating units reported a total of 39,953 women who gave birth in MUs in the UK. There were 1,673 cases of PPH requiring transfer reported, with 1,501 confirmed cases and 1,475 confirmed controls after exclusion of ineligible cases/controls and duplicates (Fig 1). Based on confirmed cases, the overall incidence of PPH requiring transfer to obstetric care was 3.7% (95% CI 3.6–3.9).

The incidence of PPH requiring transfer was significantly higher in AMUs (3.9%; 95% CI 3.6–4.1) compared with FMUs (2.6%; 95% CI 2.1–3.2) (p <0.001). Because data entry completeness was higher in AMUs (91%) compared with FMUs (80%) we also estimated incidence based on reported cases, rather than confirmed cases, but this did not materially change our results: AMU incidence 4.3%; 95% CI 4.0–4.5; FMU incidence 3.2%; 95% CI 2.7–3.8.

### Postpartum blood loss

**Fig 2:**
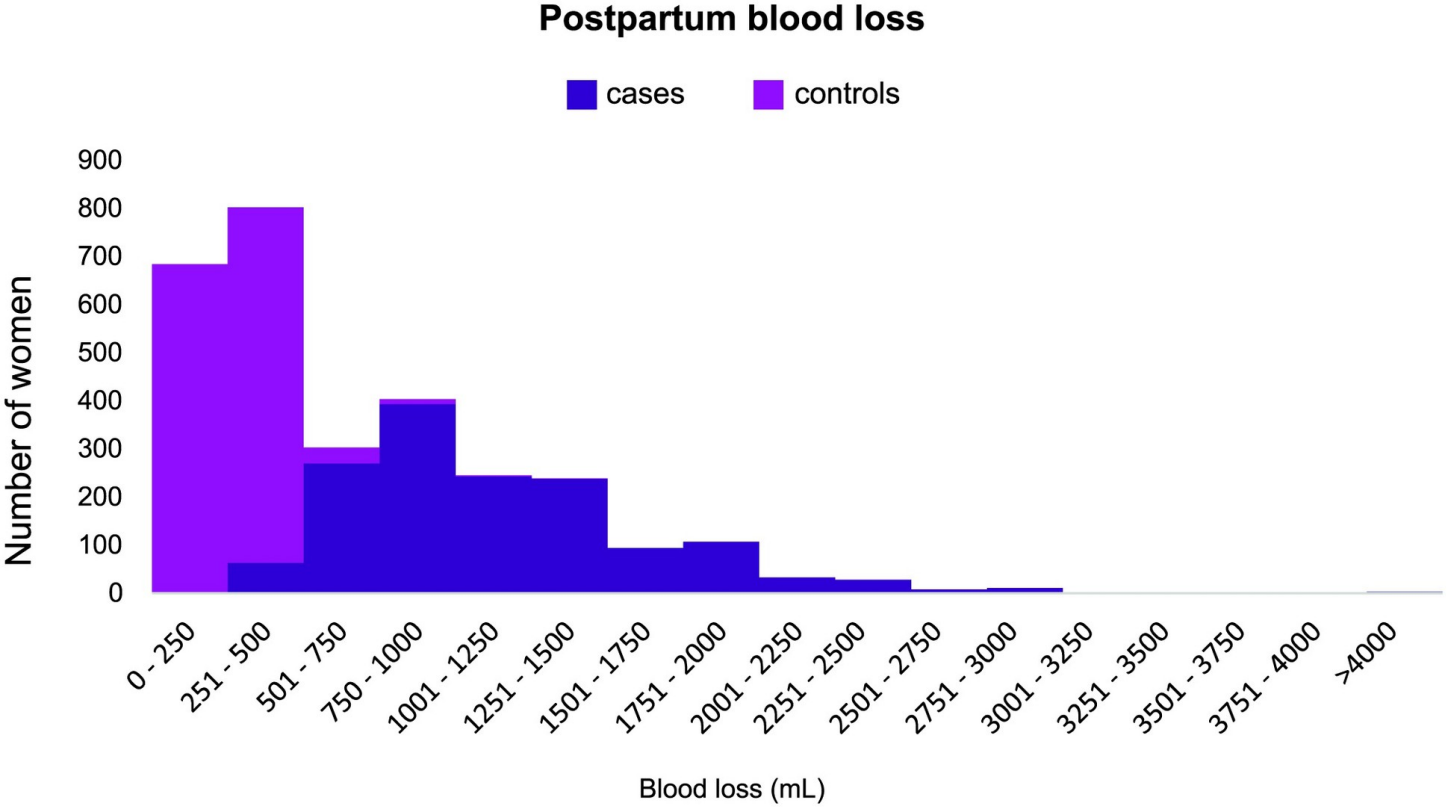
Postpartum blood loss by case/control status.

Almost all cases (95.6%) had an estimated blood loss >500mL (range: 400-5500mL; median: 1050mL; IQR 700-1450mL) while most controls (93.4%) had an estimated blood loss ≤500mL (range: 20-1300mL; median: 300mL IQR: 200-380mL) (Table 1, Fig 2). There were 92 controls (6.2%) who had an estimated blood loss ≥500mL (Table 1); PPH for these control women was managed by midwives in the MU without transfer to obstetric care.

**Table 1:** Postpartum blood loss among cases and controls.

	Controls n = 1475		Cases n = 1501	
	n	%	n	%
**Blood loss (mL)**				
<500	1377	93.4	<5	<0.3
500	46	3.1	61	4.1
501–999	43	2.9	554	36.9
1000–1499	3	0.2	517	34.4
≥1500	0	0.0	363	24.2
Missing	6	0.5	<5	<0.3

### Risk factors for PPH requiring transfer

In the univariable analysis, the sociodemographic, pre-existing clinical and pregnancy-related factors significantly associated with PPH requiring transfer were: not smoking, socioeconomic status, nulliparity, previous PPH, previous pregnancy complication other than PPH, and gestational age of 41 weeks’ or more (Table 2).

**Table 2:** Sociodemographic, pre-existing and pregnancy-related characteristics of women.

	Controls (n = 1475)		Cases (n = 1501)		Unadjusted ORs		p value
	n	%	n	%	OR	95% CI	
**Maternal age**							0.68
<20	50	3.4	48	3.2	0.98	(0.66–1.46)	
20–24	216	14.6	205	13.7	0.97	(0.75–1.25)	
25–29	427	28.9	418	28.1	1	.	
30–34	498	33.8	552	36.7	1.13	(0.93–1.38)	
35–40	260	17.6	253	16.6	0.99	(0.77–1.27)	
>40	24	1.6	25	1.6	1.06	(0.61–1.85)	
Missing	0	.	0	.	.	.	
**Smoking status**							0.005
Did not smoke during pregnancy	1274	8.4	1351	90.0	1	.	
Smoked during pregnancy	171	11.6	121	8.1	0.67	(0.52–0.85)	
Missing	30	2.0	29	1.9	0.91	(0.62–1.34)	
**Area-based deprivation quintile**							0.13
1st (least deprived)	321	21.9	380	25.5	1	.	
2^nd^	312	21.3	201	20.2	0.81	(0.67–0.99)	
3^rd^	297	20.3	270	18.1	0.77	(0.62–0.95)	
4^th^	276	18.8	274	18.4	0.84	(0.68–1.03)	
5^th^ (most deprived)	260	17.7	265	17.8	0.86	(0.69–1.06)	
Missing	10	.	11	.	.	.	.
**Ethnicity**							0.94
White (UK and Ireland)	986	66.9	997	66.4	1	.	
White (other)	182	12.3	189	12.6	1.03	(0.82–1.29)	
Asian	156	10.6	171	11.4	1.08	(0.85–1.38)	
Black	64	4.3	64	4.3	1.00	(0.70–1.39)	
Other	87	5.9	80	5.3	0.91	(0.63–1.31)	
Missing	0	.	0	.	.	.	
**Socioeconomic status**							0.010
Higher managerial	428	29.0	526	35.0	1	.	
Intermediate	289	19.6	264	17.6	0.75	(0.61–0.91)	
Routine and manual	358	24.3	335	22.3	0.76	(0.64–0.91)	
Employed, occupation unknown	104	7.1	103	6.9	0.81	(0.60–1.08)	
Unemployed/ student	101	6.9	80	5.3	0.65	(0.47–0.90)	
Not recorded	195	13.2	193	12.9	0.80	(0.66–0.98)	
**BMI at booking**							0.73
< 18.5	48	3.3	42	2.8	0.88	(0.57–1.37)	
18.5–24.9	797	54.0	789	52.6	1	.	
25–29.9	406	27.5	432	28.8	1.07	(0.91–1.27)	
30–35	153	10.4	168	11.2	1.11	(0.88–1.40)	
>35	28	1.9	30	2.0	1.08	(0.62–1.88)	
BMI not recorded	43	2.9	40	2.7	0.94	(0.65–1.37)	
**Parityº**							< .001
0	513	34.8	750	50.0	1.74	(1.51–2.02)	
1	654	44.3	548	36.5	1	.	
2 or more	308	20.9	203	13.5	0.79	(0.64–0.97)	
Missing	0	.	0	.	.	.	
**Pre-existing medical risk factors** *							0.52
No pre-existing medical risk factors	1453	98.5	1474	98.2	1	.	
One or more	22	1.5	27	1.8	1.21	(0.67–2.17)	
Missing	0	.	0	.	.	.	
**Previous pregnancy complications** **							<0.001
No previous complication	909	94.5	650	86.6	1	.	
Previous PPH	37	3.9	74	9.9	2.80	(1.77–4.41)	
Previous complication other than PPH	16	1.7	27	3.6	2.36	(1.33–4.17)	
**Current pregnancy problems º**							0.79
None	1382	93.7	1403	93.5	1	.	
One or more	93	6.3	98	6.5	1.04	(0.77–1.39)	
Missing	0	.	0	.	.	.	
**Sex of baby**							0.93
Male	710	48.3	717	47.9	1.01	(0.88–1.18)	
Female	761	51.7	781	52.1	1	.	
Missing	4	.	3	.	.	.	
**Gestational age (weeks)**							<0.001
36–37	66	4.5	38	2.5	0.58	(0.37–0.91)	
38	167	11.3	143	9.4	0.86	(0.67–1.09)	
39	399	27.1	358	23.9	0.90	(0.77–1.05)	
40	591	40.1	589	39.2	1	.	
41–43	251	17.0	373	24.9	1.49	(1.21–1.83)	
Missing	1	.	0	.	.	.	

º Number of previous pregnancies carried to at least 24 completed weeks’ gestation

* Hypertension, confirmed cardiac disease, thromboembolic disorder, atypical antibodies, hyperthyroidism, diabetes, renal disease, epilepsy.

**Includes all women with a PPH including those who also had another previous pregnancy problem. Previous complications were: Retained placenta requiring manual removal and Caesarean section. Excludes primiparous women.

^†^ Retained placenta requiring manual removal, Caesarean section, Uterine surgery excluding Caesarean section and shoulder dystocia. Excludes primiparous women.

**º** BMI at booking >35, Post-term (>42 weeks) Anaemia, Group B Streptococcus, Antepartum haemorrhage, Pre-eclampsia/pregnancy-induced hypertension, Gestational diabetes, Malpresentation (breech or transverse lie), multiple pregnancy

The intrapartum and birth-related factors significantly associated with PPH requiring transfer in the univariable analysis were: induction of labour, immersion in water during labour, maternal complications identified at the start of labour care, fetal complications identified at the start of labour care, maternal complications identified during labour, instrumental vaginal delivery, a third stage of labour ≥60 minutes, perineal trauma, Syntocinon/ Syntometrine for 3rd stage management, and birthweight ≥3500g (Table 3).

**Table 3:** Intrapartum and birth-related factors.

	Controls (n = 1475)		Cases (n = 1501)		Unadjusted ORs		p value
	n	%	n	%	OR	95% CI	
**Stage of labour at start of care**							0.63
Latent stage	276	18.7	270	18.1	0.94	(0.77–1.15)	
Active 1st stage	1030	70.0	1072	71.7	1	.	
Passive 2nd stage	53	3.6	46	3.1	0.83	(0.58–1.20)	
Active 2nd stage	113	7.7	108	7.2	0.92	(0.69–1.22)	
Missing	3	.	5	.	.	.	
**Induction of labour**							0.022
No	1412	96.2	1410	94.4	1	.	
Yes	56	3.8	84	5.6	1.50	(1.06–2.13)	
Missing	7	.	7	.	.	.	
**Maternal complications identified at the start of labour care** *							0.003
None	1455	98.9	1457	97.5	1	.	
One or more	16	1.1	38	2.5	2.37	(1.33–4.23)	
Missing	4	.	6	.			
**Fetal complications identified at the start of labour care** †							0.008
None	1443	98.1	1446	96.7	1	.	
One or more	28	1.9	49	3.3	1.75	(1.15–2.64)	
Missing	4	.	6	.	.	.	
**Maternal complications identified during labour (before birth)** º							0.007
None	1451	98.4	1458	97.2	1	.	
One or more	24	1.6	43	2.8	1.78	(1.17–2.71)	
Missing	0		0				
**Fetal complications identified during labour (before birth)** ^¶^							0.005
None	1376	93.2	1360	90.6	1		
One or more	99	6.8	141	9.4	1.44	(1.12–1.86)	
Missing	0		0				
**Labour/birth in water**							0.004
No immersion in water	820	55.7	808	54.0	1	.	
Immersion in water for labour, land birth	217	14.7	263	17.6	1.23	(1.04–1.45)	
Birth in water	435	29.6	425	28.4	0.99	(0.86–1.14)	
Missing	3	.	5	.	.	.	
**Birth mode**							<0.001
Spontaneous vertex birth	1459	99.2	1456	97.7	1	.	
Vaginal breech	<5	<0.3	0	0	.	.	
Instrumental	10	0.7	35	2.4	3.51	(2.25–5.43)	
Missing	4	.	10	.	.	.	
**Duration of third stage of labour**							<0.001
< 60 minutes	1399	94.9	1240	82.6	1	.	
≥ 60 minutes	53	3.6	227	15.1	4.83	(3.43–6.79)	
Missing	23	1.6	34	2.3	.	.	
**Perineal trauma**							<0.001
<3^rd^ degree tear or no tear	1427	96.9	1290	86.2	1	.	
3^rd^ or 4^th^ degree tear	45	3.1	207	13.8	5.09	(3.49–7.42)	
Missing	3	.	4	.	.	.	
**Syntocinon/ Syntometrine for 3rd stage management**							0.001
No	278	18.9	205	13.7	1		
Yes	1194	81.1	1291	86.3	1.47	(1.17–1.83)	
Missing	4	.	5	.	.	.	
**Birthweight (g)**							<0.001
<3000	192	13.1	112	7.5	0.74	(0.57–0.96)	
3000–3499	644	43.8	508	33.9	1	.	
3500–3999	485	33.0	628	41.9	1.64	(1.37–1.96)	
≥4000	150	10.2	250	16.7	2.11	(1.66–2.69)	
Missing	4	.	3	.	.	.	

* Maternal tachycardia, hypertension, proteinuria, maternal pyrexia, vaginal blood loss, prolonged rupture of membranes, and reported pain differing from pain normally associated with contractions.

† Significant meconium, abnormal presentation, high or free-floating head, suspected anhydramnios or polyhydramnios, fetal heart rate abnormality, deceleration in fetal heart rate, and reduced fetal movements in the last 24 hours.

º Maternal tachycardia, hypertension, maternal prexia, vaginal blood loss, prolonged rupture of membranes.

^¶^ Significant meconium, confirmed/suspected delay in first stage of labour, confirmed\suspected delay in second stage of labour, obstetric emergency, abnormal presentation, fetal heart rate abnormality, and deceleration in fetal heart rate.

Multivariable analysis indicated that, compared with controls, women who experienced a PPH requiring transfer to obstetric care were less likely to have smoked during pregnancy (aOR = 0.73; 95% CI 0.56–0.94) (Table 4). They were also more likely to be giving birth for the first time (aOR = 1.96; 95% CI 1.66–2.30); to have experienced a previous PPH (aOR = 2.67; 95% CI 1.67–4.25) or another complication in a previous pregnancy if they had given birth before (aOR = 2.40; 95% CI 1.25–4.60); to be giving birth at or after 41 weeks’ gestation (aOR = 1.36; 95% CI 1.10–1.69). In term of risk factors related to the birth, women who had a PPH requiring transfer to obstetric care were more likely to have an instrumental birth (aOR = 2.69 95% CI 1.53–4.72); to have a third stage of labour lasting 60 minutes or longer (aOR = 5.56 95% CI 3.93–7.88); to experience a third or fourth degree perineal tear (aOR = 4.67 95% CI 3.16–6.90); or to give birth to a baby weighing 3500-3999g (aOR = 1.71 95% CI 1.42–2.07), or >4000g (aOR = 2.31 95% CI 1.78–3.00).

**Table 4:** Independent factors associated with PPH in midwifery units requiring transfer to obstetric care.

	Controls		Cases		Unadjusted analysis		Adjusted analysis n = 2940		
	n	%	n	%	OR	(95% CI)	aOR	(95% CI)	p value
**Smoking status**									0.031
Did not smoke during pregnancy	1274	86.4	1351	90.0	1	.	1		
Smoked during pregnancy	171	11.6	121	8.1	0.66	(0.52–0.85)	0.73	(0.56–0.94)	
Missing	30	2.0	29	1.9	0.91	(0.62–1.34)	0.80	(0.48–1.31)	
**Area-based deprivation quintile**									0.019
1st (least deprived)	321	21.9	380	25.5	1	.	1		
2^nd^	312	21.3	201	20.2	0.81	(0.67–0.88)	0.87	(0.71–1.07)	
3^rd^	297	20.3	270	18.1	0.77	(0.62–0.95)	0.88	(0.71–1.09)	
4^th^	276	18.8	274	18.4	0.84	(0.68–1.03)	1.04	(0.83–1.31)	
5^th^ (most deprived)	260	17.7	265	17.8	0.86	(0.69–1.07)	1.27	(1.00–1.61)	
**Parity**									<0.001
0	513	34.8	750	50.0	1.87	(1.62–2.15)	1.96	(1.66–2.30)	
1	654	44.3	548	36.5	1	.	1		
2+	308	20.8	203	13.5	0.78	(0.64–0.97)	0.81	(0.65–1.01)	
**Previous pregnancy complication** *									
No previous complication	909	94.5	650	86.6	1	.	1	.	
Previous PPH	37	3.9	74	9.9	2.80	(1.77–4.41)	2.67	(1.67–4.25)	<0.001
Previous complication other than PPH	16	1.7	27	3.6	2.36	(1.33–4.17)	2.40	(1.25–4.60)	0.009
**Gestational age**									0.007
36–37	66	4.5	38	2.5	0.58	(0.37–0.91)	0.69	(0.42–1.14)	
38	167	11.3	143	9.4	0.86	(0.68–1.10)	1.04	(0.80–1.36)	
39	399	27.1	358	23.9	0.90	(0.77–1.05)	0.98	(0.82–1.17)	
40	591	40.1	589	39.2	1	.	1	.	
41–43	251	17.0	373	24.9	1.49	(1.21–1.84)	1.36	(1.10–1.69)	
**Birth mode**									<0.001
Spontaneous vertex or vaginal breech birth	1461	99.2	1456	97.7	1	.	1	.	
Instrumental birth	10	0.82	35	2.35	2.92	(1.83–4.64)	2.69	(1.53–4.72)	
**Duration of third stage of labour**									<0.001
< 60 minutes	1399	94.9	1240	82.6	1	.	1	.	
≥ 60 minutes	53	3.6	227	15.1	4.8	(3.42–6.81)	5.56	(3.93–7.88)	
Missing	23	1.6	34	2.3	.	.	.	.	
**Perineal tear**									<0.001
<3rd degree tear or no tear	1427	96.9	1290	86.2	1	.	1	.	
3rd or 4th degree tear	45	3.1	207	13.8	5.09	(3.49–7.42)	4.67	(3.16–6.90)	
**Birthweight (gm)**									<0.001
<3000	192	13.1	112	7.5	0.47	(0.57–0.96)	0.78	(0.57–1.06)	
3000–3499	644	43.8	508	33.9	1	.	1	.	
3500–3999	485	33.0	628	41.9	1.64	(1.37–1.96)	1.71	(1.42–2.07)	
≥4000	150	10.2	250	16.7	2.11	(1.66–2.69)	2.31	(1.78–3.00)	

º Each variable in the model is adjusted for all other variables in the model. Includes 2,940 observations

* Excludes primiparous women

### Maternal and neonatal outcomes following a PPH

Overall, 158 cases (11%) received a transfusion of blood or blood products following their PPH (Table 5). One in five cases (21%) was admitted to higher level care, compared with <0.3% of controls. Five cases and no controls were admitted to intensive care. Of those admitted to higher level care, less than 2% of cases and no controls stayed longer than two days in higher level care. Initiation of breastfeeding before discharge was similar among cases (82%) and controls (80%).

**Table 5:** Maternal outcomes among cases and controls.

	Controls n = 1475		Cases n = 1501	
	n	%	n	%
**Blood transfusion**	na	na	158	10.6
**Maternal morbidity reported** *	<5	<0.3	30	2.0
**Admission to higher level of care**	<5	<0.3	308	21.0
**Type of higher level care** †				
Enhanced maternity care	<5	100.0	303	98.3
Intensive care	0	0	5	1.6
**Duration of stay in higher level care** †				
< 1 day	0	0	<5	<1.6
1 day	<5	na	269	88.8
2 days	<5	na	26	8.6
3 or more days	0	0	<5	<1.6
Missing	0	0	5	.
**Primary reason for higher level care** †				
PPH and observation following PPH	0	0.0	232	74.3
Observation (without PPH specified)	0	0	42	14.0
Recovery from theatre procedure	<5	<0.3	12	3.6
Sepsis/pyrexia & Other/not clear	<5	<0.3	22	7.1
**Any ‘enhanced treatment or care’** ^¶^	7	0.6	370	24.7
**Breastfeeding initiated before discharge**				
Yes	1173	79.7	1221	81.5
No	298	20.3	276	18.4
Missing	4	.	4	.

* Maternal morbidity includes: readmission for secondary PPH/retained placenta, pyrexia/sepsis, anaemia and ‘other’ morbidities

^**†**^ Proportions are among women admitted to higher level care

^¶^ Presence of either of the following: blood transfusion, admission to higher level-care

One in four cases (25%) received ‘enhanced treatment or care’, compared with 0.6% of controls. Most women who received ‘enhanced treatment or care’ (79%) were admitted for higher level care, primarily for observation following PPH. There were no maternal deaths among cases or controls in this study.

Among women who had a PPH requiring transfer, those who received ‘enhanced treatment or care’ were not significantly different from those who did not in terms of maternal sociodemographic characteristics, pre-existing clinical risk factors or pregnancy-related factors (S1–S5 Tables).

The only labour- and birth-related factor associated with an increased risk of ‘enhanced treatment or care’ was duration of third stage of labour lasting 60 minutes or longer (OR 1.14 95% CI 1.34–2.41) (Table 6). Postpartum blood loss of 1000-1499mL was associated with higher odds of receiving ‘enhanced treatment or care’ (OR 5.53; 95% CI 1.70–17.79), as was blood loss of 1500mL or more (OR 33.0 95% CI 10.15–107.24), compared with blood loss of 500mL or less. Genital tract trauma as the primary cause of the PPH was associated with lower odds of ‘enhanced treatment or care’ (OR 0.58 95% CI 0.43–0.79).

**Table 6:** Univariable analysis of risk factors for ‘enhanced treatment or care’ following PPH requiring transfer.

	No ‘enhanced treatment or care’ (n = 1,131)		‘Enhanced treatment or care’ (n = 370)		Unadjusted ORs		p value
	n	%	n	%	OR	95% CI	
**Duration of third stage of labour**							<0.001
< 60 minutes	956	84.5	284	76.8	1	.	
≥ 60 minutes	148	13.1	79	21.4	1.79	(1.34–2.41)	
Missing	27	2.4	7	1.9	0.87	(0.40–1.92)	
**Cause of PPH**							<0.001
Uterine atony	406	35,9	152	41.1	1	.	
Genital tract trauma	355	31.4	77	20.8	0.58	(0.42–0.80)	
Retained products / morbidly adherent placenta	221	19.5	106	28.7	1.28	(0.95–1.73)	
Other	15	1.3	6	1.6	1.07	(0.45–2.52)	
Not recorded	134	11.9	29	7.8	0.59	(0.36–0.94)	
**Blood loss (mL)**							<0.001
≤500	60	5.3	<5	<1.2	0.91	(0.24–3.42)	
501–999	525	46.4	29	7.8	1	.	
1000–1499	405	35.8	112	30.3	5.01	(3.06–8.18)	
1500	137	12.1	226	61.1	33.0	(17.14–52.02)	
Missing	4	.	<5	<0.3	.	.	

Neonatal outcomes were generally good, with small numbers of babies reported as having a neonatal morbidity, Apgar score <7 at 5 minutes or admission to higher level care. These neonatal outcomes were slightly more common among babies born to women who had a PPH requiring transfer to obstetric care (S6 Table). Two babies died in the neonatal/post-neonatal period. There were no stillbirths in this study.

### Prevalence of risk factors among women giving birth in AMUs and FMUs

The prevalence of risk factors for PPH requiring transfer among cases and controls was similar in AMUs and FMUs (S7 and S8 Tables). There was no statistically significant difference between FMUs and AMUs in terms of blood loss volume among cases or controls (S9 Table).

## Discussion

This study provides valuable information about the incidence of and risk factors for PPH occurring in MUs in the UK, to support midwifery practice and women’s decision-making. Among women giving birth in UK MUs, the estimated incidence of PPH requiring transfer to obstetric care was 3.7%. Several independent risk factors for PPH requiring transfer were identified through multivariable analysis. Of the risk factors that are known prior to admission for the birth, primiparity, not smoking in pregnancy, previous PPH, problems in a previous pregnancy other than PPH, and gestational age ≥41 weeks were associated with higher odds of having a PPH requiring transfer to obstetric care. The factors occurring during labour and birth associated with higher odds of having a PPH requiring transfer were instrumental birth, a third stage of labour lasting ≥60 minutes, 3^rd^ or 4^th^ perineal tear, and birthweight ≥3500g.

This study also provides information about outcomes following a PPH in an MU in the UK. Among women who had a PPH requiring transfer to obstetric care, a third stage of labour lasting 60 minutes or longer was associated with higher odds of requiring ‘enhanced treatment or care’, while genital tract trauma was associated with lower odds of requiring ‘enhanced treatment or care’.

The incidence of PPH requiring transfer to obstetric care, following birth in a midwifery unit, was higher than we anticipated. There are, however, few data against which to compare our estimates. In the Birthplace national prospective cohort study, which investigated outcomes by planned place of birth in England in 2008–10, the proportion of women who were transferred from an MU to obstetric care where the primary reason for transfer was PPH was 1.0% in FMUs and 1.0% in AMUs [11]. However, these results may not be directly comparable as they only included women whose **primary** reason for transfer was PPH, whereas our study also included women for whom PPH was a secondary reason for transfer. The Birthplace study was carried out in 2008–10. Since then, the overall proportion of women experiencing a PPH in the UK has also increased, as it has in other high-income countries [6, 19, 20]. Between 2010 and 2021, the rate of PPH among spontaneous vaginal births in England more than doubled, from 7.2% to 16.0% [21, 22]. While these data relate to births in all NHS hospitals and thus are not directly comparable to births solely in MUs, it is possible that this general upward trend may also be seen in women giving birth in an MU. Research into the incidence of PPH in other high-income countries suggests that the recent increased incidence does not appear to be entirely explained by increasing prevalence of risk factors in the population [6, 19, 20, 23], which suggests that the incidence of PPH may have also increased among ‘lower risk’ women who give birth in MUs.

The incidence of PPH requiring transfer was higher in AMUs (3.9%) compared with FMUs (2.6%). Typically, women planning birth in FMUs have fewer pre-existing and current pregnancy complications compared with women who plan birth in an AMU [11], but our further analysis comparing the characteristics of women giving birth in the two types of unit indicated that this is unlikely to explain the difference in incidence we found. Our case definition for this study was ‘PPH requiring transfer’, rather than PPH, so this is likely to have been influenced by midwives’ decision-making or ‘threshold’ for transfer. It is also possible therefore that the higher incidence of PPH requiring transfer in AMUs, compared with FMUs, may be explained by more readily-available access to obstetric care in AMUs. Midwives in AMUs might, as a consequence, have a lower ‘threshold’ for transfer for PPH, particularly for women whose blood loss might be managed under midwifery care, i.e. those with smaller amounts of blood loss. However, our post hoc analysis revealed that postpartum blood loss volume was similar in FMUs and AMUs among both cases and controls, suggesting that there were not significant numbers of women whose PPH was managed without transfer in FMUs.

The risk factors for PPH identified in this study broadly align with the few previous studies of PPH in populations of women considered at low risk of complications [24–26]. Smoking status was significantly but inversely associated with PPH requiring transfer to obstetric care, with women who smoked during pregnancy being almost 30% less likely to experience a PPH requiring transfer than those who did not smoke. The hypercoagulation effects of smoking may contribute to this association [27], but it is also possible that the observed association between not smoking and PPH requiring transfer is a result of residual confounding in our study. The literature about this association is inconclusive, with some studies reporting that smoking may be a protective factor for PPH [28–30] and others indicating it might be a risk factor [31, 32] suggesting that further research is required to investigate this association.

In contrast to some previous research [28, 33, 34], we did not find ethnicity to be significantly associated with PPH. A study using routine clinical data about more than 900,000 women giving birth in maternity units in England from 2015–17 found that women from ethnic minority backgrounds had an increased risk of severe PPH, after adjusting for some maternal, fetal and birth factors [34]. In our study, while the distribution of ethnicity overall was similar, the lack of association between ethnicity and PPH may be explained by the selected ‘low risk’ population giving birth in MUs.

Consistent with previous studies [33, 35, 36], we found strong evidence for nulliparity as a risk factor for PPH. Women who had not previously given birth were almost twice as likely to have a PPH requiring transfer, compared with women who had given birth once before. Women who give birth in MUs in the UK are more likely to have given birth before than be giving birth for the first time [11], and this was reflected in our study, with 65% of controls and 50% of cases being multiparous. While the risk of PPH among primiparous women in our study was higher than in women who had given birth before, the absolute risk of PPH following birth in a MU remains low for primiparous women giving birth in MU. Among all women giving birth in England, primiparous women have higher rates of PPH, compared with women who have given birth previously [22]. This, combined with the observation that primiparous women who had a PPH requiring transfer were not at higher risk of requiring ‘enhanced treatment or care’ compared with multiparous women, suggests that overall nulliparous women who are eligible to give birth in a MU should consider this option.

National guidance recommends that women who have had previous pregnancy complications, including a previous PPH requiring treatment or transfusion, should be advised to plan birth in obstetric units in the UK [3]. However, local NHS guidance about admission criteria for midwifery-led care varies widely from national guidance [8]. A national survey, carried out in 2018–19, found that in most MUs whose admission criteria explicitly mentioned PPH these criteria were not in alignment with current guidelines, with 1 in 4 (27%) admission guidelines that mentioned PPH as an admission criterion using previous blood loss <1L as the stated inclusion criteria, and 5% admitting women with a previous PPH <1.5L or <2L [8]. In our study, a small proportion of women had a previous PPH requiring treatment or transfusion (10% of cases and 4% of controls) and a smaller proportion had previous complications other than PPH (4% of cases and 2% of controls), including for example previous retained placenta requiring manual removal or previous caesarean section. Among women who had given birth at least once before, previous PPH requiring treatment or blood transfusion increased a woman’s odds of PPH requiring transfer by 2.7 times. Women who had a previous pregnancy complication other than PPH were 2.4 times more likely to have a PPH requiring transfer. Previous retained placenta requiring manual removal was the most common previous complication other than PPH, accounting for 70% of the cases with previous complications.

Several other intrapartum factors, including induction, immersion in water during labour, and complications identified during labour, were associated with increased odds of PPH requiring transfer at the univariable level, but not after adjustment for other factors. With the exception of immersion in water during labour, the proportion of women with these risk factors was relatively low, with very small numbers of women affected by each individual complication. Our study confirms advanced gestational age as an independent risk factor for PPH [31, 37–39]; women who gave birth at 41–42 weeks’ were over 30% more likely to have a PPH requiring transfer, compared with women giving birth at 40 weeks’ gestation.

In line with previous research, instrumental birth was associated with a 2.7 fold increase in the odds of PPH requiring transfer to obstetric care. Instrumental birth occurs only infrequently in MUs; in our study, 2.4% of cases and 0.7% of controls had an instrumental birth. It should be noted that this does not reflect the proportion of women who plan birth in a MU and have an instrumental birth, as women who were transferred from an MU to OU before the birth were not included in our study. Instrumental births in MUs are only performed in AMUs and almost always in circumstances in which expediting birth is a priority over physically transferring the woman to the obstetric unit. For women who do have an instrumental birth in a MU, our study suggests that vigilance by midwives following an instrumental birth is important.

The most significant independent risk factor for PPH requiring transfer was a third stage of labour lasting ≥60 minutes, which increased a woman’s risk of PPH more than fivefold. This is consistent with previous research indicating that a longer third stage of labour is associated with an increased risk of PPH [40–42]. A prolonged third stage of labour can contribute to higher amounts of blood loss due to prolonged bleeding from the placental site and from unrepaired perineal trauma [43]. UK national guidance recommends that a third stage of labour should be diagnosed as prolonged if it lasts longer than 30 minutes with active management, or longer than 60 minutes with physiological management. Data were collected about the administration of syntocinon or syntometrine for active management of the third stage of labour, but with the available data it was not possible to determine whether syntocinon or syntometrine were administered as part of **planned** active management of labour or whether administration was indicated because of increased blood loss. Such information may have strengthened our analysis of the risk associated with duration of the third stage of labour.

Women who had a PPH requiring transfer were more likely to require ‘enhanced treatment or care’ (comprising admission to higher level care or blood transfusion) after birth, compared with controls (24.7% vs 0.6%). Among cases, women who required ‘enhanced treatment or care’ were not significantly different from women who did not, in terms of sociodemographic, clinical or pregnancy-related factors. This suggests it would not be feasible for midwives to identify women likely to be in need of enhanced care following PPH based on pre-identified characteristics. However, it is possible that the study was underpowered to detect differences in these potential risk factors because there were only small numbers of women in the ‘enhanced treatment or care’ group. The risk factors for enhanced treatment were more proximal labour- and PPH-related factors, including duration of the third stage of labour, cause of PPH and blood loss volume.

In our study, in a generally low risk population, 3.7% of births in midwifery units were affected by a PPH requiring transfer to obstetric care, with 1 in 4 cases having a reported blood loss of 1500mL or greater. This represents significant potential for maternal morbidity. However, the broadly positive outcomes for women following PPH in an MU, evidenced in this study, appear indicative of appropriate management. The most common reason for admission to higher-level care was for observation following the PPH, which was indicated in some free text comments to be standard practice. Most women (90%) who were admitted to higher-level care stayed for less than two days, and only five (1.6%) were admitted to the ICU. Some pre-existing risk factors, including previous PPH and other previous pregnancy complications, were significantly associated with an increased risk of PPH requiring transfer, and there were indications at the univariable level that some complications arising during labour were also associated with an increased risk. There is no evidence however that planned birth in an OU or transfer to an OU prior to birth, for example for women with identified or emerging risk factors, would have either reduced the risk of having a PPH or improved outcomes following a PPH. There is also strong evidence that for women a positive birth experience includes clinical safety and psychosocial wellbeing, including involvement in decision-making, and care that is in line with their values and preferences[44], so women’s choice, supported by appropriate evidence, is also important. In this context, to maintain safe care for women planning birth in MUs it is imperative that NHS organisations have robust guidelines about the management of PPH in MUs, appropriate equipment and training[45] and ready access to transfer when required.

### Strengths and limitations

A major strength of this study was its robust design; a national population-based case-control study which included all reported cases of PPH requiring transfer to obstetric care following birth in an MU. The inclusion of all MUs in the UK in the study and 98% participation rate minimises the risk of bias related to regional differences between MUs across the UK. Additionally, the high monthly reporting rate, with 95% response rate to monthly requests for data, reduced the likelihood of selection bias.

The case definition used in this study was based on the decision to transfer a woman to obstetric care, rather than on a specified volume of postpartum blood loss. This definition was chosen to capture women whose condition was considered severe enough to warrant transfer to obstetric care, rather than using estimated blood loss. At the time of data collection, visual estimation of blood loss was typically used in MUs, which previous studies have shown to be unreliable [14, 15], although quantitative measurement of blood loss is becoming more common [13]. Almost all cases had an estimated blood loss >500mL, the definition of PPH used in the UK [46], and almost all controls had an estimated blood loss ≤500mL. This suggests that the case definition employed here is comparable to other studies that use estimated blood loss of >500mL to define cases of PPH. The decision to transfer a woman for PPH may have been influenced by the resources and capacity in the MU and the OU at the time of the birth. Free text comments entered by some reporting midwives indicated that the capacity of both MUs and OUs may have influenced the decision to transfer a woman, or not, following a PPH. Because we were reliant on anonymised data entered directly from medical records, we did not have data on several factors of interest that might have shed more light on this including, for example, staffing levels in the MU and OU around the time of the birth.

This study was planned to run for 12 months but was cut short due to the COVID-19 pandemic. Had data collection been able to proceed as planned, the study would have had greater power as planned to be able to detect associations between PPH and putative risk factors that were uncommon in this population.

## Conclusions

PPH requiring transfer to obstetric care following birth in an MU is a relatively uncommon event in the UK, but incidence may be increasing. The risk factors associated with the most significant increase in the odds of a PPH requiring transfer to obstetric care were a third stage of labour lasting 60 minutes or more, and perineal trauma. Our results about outcomes for women who have a PPH in an MU are broadly reassuring and indicative of appropriate management. It remains important that NHS organisations have robust guidelines about the management of PPH in MUs, appropriate equipment and training, and ready access to transfer when required.

## Supporting information

S1 Table Maternal sociodemographic characteristics among women who had a PPH requiring transfer, according to whether they received ‘enhanced treatment or care’. (DOCX)

S2 Table Pre-existing clinical characteristics among women who had a PPH requiring transfer, according to whether they received ‘enhanced treatment or care’. (DOCX)

S3 Table Clinical characteristics arising during pregnancy among women who received enhanced care and women who did not receive enhanced care. (DOCX)

S4 Table Intrapartum factors among women who received enhanced care and women who did not receive enhanced care. (DOCX)

S5 Table Birth-related factors among women who received enhanced care and women who did not receive enhanced care. (DOCX)

S6 Table Neonatal outcomes among cases and controls. (DOCX)

S7 Table Risk factors for PPH requiring transfer to obstetric care among cases according to the type on unit in which they gave birth. (DOCX)

S8 Table Risk factors for PPH requiring transfer to obstetric care among controls according to the type on unit in which they gave birth. (DOCX)

S9 Table Blood loss among cases and controls by unit type. (DOCX)

## References

[1] NMPA. National Maternity and Perinatal Audit: Clinical Report 2017. London; 2017.

[2] Department of Health. Changing childbirth: Part I: Report of the Expert Maternity Group. London: HMSO; 1993.

[3] (NICE) NIfHaCE. Intrapartum Care: Care of Healthy Women and their Babies During Childbirth. London; 2014. 25950072

[4] Endo-Kawamura N, Obata-Yasuoka M, Yagi H, Ohara R, Nagai Y, Mayumi M, et al. Higher D-dimer level in the early third trimester predicts the occurrence of postpartum hemorrhage. Journal of Perinatal Medicine. 2016;44(5):551–6. doi: 10.1515/jpm-2015-0287 26756085

[5] Bonnet MP, Basso O, Bouvier-Colle MH, Dupont C, Rudigoz RC, Fuhrer R, et al. Postpartum haemorrhage in Canada and France: a population-based comparison. PLoS ONE [Electronic Resource]. 2013;8(6):e66882. doi: 10.1371/journal.pone.0066882 23826165 PMC3691240

[6] Kaelin Agten A, Passweg D, von Orelli S, Ringel N, Tschudi R, Tutschek B. Temporal trends of postpartum haemorrhage in Switzerland: a 22-year retrospective population-based cohort study. Swiss Medical Weekly. 2017;147:w14551. doi: 10.4414/smw.2017.14551 29185249

[7] Looft E, Simic M, Ahlberg M, Snowden JM, Cheng YW, Stephansson O. Duration of Second Stage of Labour at Term and Pushing Time: Risk Factors for Postpartum Haemorrhage. Paediatric and Perinatal Epidemiology. 2017;31(2):126–33. doi: 10.1111/ppe.12344 28195653

[8] Glenister C, Burns E, Rowe R. Local guidelines for admission to UK midwifery units compared with national guidance: A national survey using the UK Midwifery Study System (UKMidSS). PloS one. 2020;15(10):e0239311–e. doi: 10.1371/journal.pone.0239311 33079940 PMC7575094

[9] Rowe R, Knight M, Kurinczuk JJ. Outcomes for women with BMI>35kg/m2 admitted for labour care to alongside midwifery units in the UK: A national prospective cohort study using the UK Midwifery Study System (UKMidSS). PloS one. 2018;13(12):e0208041–e. 30513088 10.1371/journal.pone.0208041 PMC6279017

[10] Morelli A, Rogers J, Sanders J, Kurinczuk JJ, Rowe R. Outcomes for women admitted for labour care to alongside midwifery units in the UK following a postpartum haemorrhage in a previous pregnancy: A national population-based cohort and nested case-control study using the UK Midwifery Study System (UKMidSS). Women and birth: journal of the Australian College of Midwives. 2022. 10.1016/j.wombi.2022.11.002 36376224

[11] Hollowell J, Puddicombe D, Rowe R, Linsell L, Hardy P, Stewart M, et al. The Birthplace national prospective cohort study: perinatal and maternal outcomes by planned place of birth. Birthplace in England research programme. Final report part 4. London; 2011.

[12] Rowe R, Kurinczuk J, Hollowell J, Knight M. The UK Midwifery Study System (UKMidSS): a programme of work to establish a research infrastructure to carry out national studies of uncommon conditions and events in midwifery units. BMC pregnancy and childbirth. 2016;16(1):77-. doi: 10.1186/s12884-016-0868-1 27080858 PMC4832539

[13] Bell SF, Watkins A, John M, Macgillivray E, Kitchen TL, James D, et al. Incidence of postpartum haemorrhage defined by quantitative blood loss measurement: a national cohort. BMC pregnancy and childbirth. 2020;20(1):271-. doi: 10.1186/s12884-020-02971-3 32375687 PMC7201938

[14] Sloan NL, Durocher J, Aldrich T, Blum J, Winikoff B. What measured blood loss tells us about postpartum bleeding: a systematic review. BJOG: an international journal of obstetrics and gynaecology. 2010;117(7):788–800. doi: 10.1111/j.1471-0528.2010.02567.x 20406227 PMC2878601

[15] Stafford I, Dildy GA, Clark SL, Belfort MA. Visually estimated and calculated blood loss in vaginal and cesarean delivery. American journal of obstetrics and gynecology. 2008;199(5):519.e1–.e7. doi: 10.1016/j.ajog.2008.04.049 18639209

[16] Office for National Statistics. Standard Occupational Classification 2010. Basingstoke: Palgrave Macmillan; 2010.

[17] HM Revenue & Customs. Personal tax credits: Children in low-income families local measure: 2016 snapshot as at 31 August 2016 2016 https://www.gov.uk/government/statistics/personal-tax-credits-children-in-low-income-families-local-measure-2016-snapshot-as-at-31-august-2016.

[18] Mummadi A R, Hollowell J. Risk factors of primary postpartum haemorrhage in midwifery-led setting in ‘low risk’ women: secondary analysis of the Birthplace prospective cohort study. Poster presented at 2nd European Congress on Intrapartum Care; 21–23 May 20152015.

[19] Flood M, McDonald SJ, Pollock W, Cullinane F, Davey MA. Incidence, trends and severity of primary postpartum haemorrhage in Australia: A population-based study using Victorian Perinatal Data Collection data for 764 244 births. Australian & New Zealand Journal of Obstetrics & Gynaecology. 2019;59(2):228–34. 29787638 10.1111/ajo.12826

[20] Kramer MS, Dahhou M, Vallerand D, Liston R, Joseph KS. Risk Factors for Postpartum Hemorrhage: Can We Explain the Recent Temporal Increase? Journal of obstetrics and gynaecology Canada. 2011;33(8):810–9. doi: 10.1016/S1701-2163(16)34984-2 21846436

[21] NHS Digital. NHS Maternity Statistics, 2010–11, Hospital Episode Statistics. London: NHS Digital; 2011.

[22] NHS Digital. NHS Maternity Statistics, 2020–21, Hospital Episode Statistics. London; 2021.

[23] Knight M, Callaghan WM, Berg C, Alexander S, Bouvier-Colle MH, Ford JB, et al. Trends in postpartum hemorrhage in high resource countries: a review and recommendations from the International Postpartum Hemorrhage Collaborative Group. BMC Pregnancy & Childbirth. 2009;9:55. doi: 10.1186/1471-2393-9-55 19943928 PMC2790440

[24] Nishida K, Sairenchi T, Uchiyama K, Haruyama Y, Watanabe M, Hamada H, et al. Poor uterine contractility and postpartum hemorrhage among low-risk women: A case-control study of a large-scale database from Japan. International journal of gynecology and obstetrics. 2021;154(1):17–23. doi: 10.1002/ijgo.13474 33156517

[25] Patterson JA, Nippita TA, Randall D, Irving DO, Ford JB, Bowen JR, et al. Outcomes associated with transfusion in low-risk women with obstetric haemorrhage. Vox sanguinis. 2018;113(7):678–85. doi: 10.1111/vox.12707 30159918

[26] Khireddine I, Le Ray C, Dupont C, Rudigoz R, Bouvier-Colle M, Deneux-Tharaux C. Induction of labor and risk of postpartum hemorrhage in low risk parturient women. Journal of Maternal-Fetal and Neonatal Medicine. 2012;(2):101. 10.1371/journal.pone.0054858 PMC3555986 23382990

[27] Nielsen VG, Hafner DT, Steinbrenner EB. Tobacco smoke-induced hypercoagulation in human plasma: role of carbon monoxide. Blood coagulation & fibrinolysis. 2013;24(4):405–10. doi: 10.1097/MBC.0b013e32835d5458 23429254

[28] Briley A, Seed PT, Tydeman G, Ballard H, Waterstone M, Sandall J, et al. Reporting errors, incidence and risk factors for postpartum haemorrhage and progression to severe PPH: a prospective observational study. BJOG: An International Journal of Obstetrics & Gynaecology. 2014;121(7):876–88. doi: 10.1111/1471-0528.12588 24517180 PMC4282054

[29] Oberg AS, Hernandez-Diaz S, Frisell T, Greene MF, Almqvist C, Bateman BT. Genetic contribution to postpartum haemorrhage in Swedish population: cohort study of 466,686 births. BMJ. 2014;349:g4984. doi: 10.1136/bmj.g4984 25121825 PMC4131501

[30] Parry-Smith W, Šumilo D, Subramanian A, Gokhale K, Okoth K, Gallos I, et al. Postpartum Haemorrhage and Risk of Long-Term Hypertension and Cardiovascular Disease: An English Population-Based Longitudinal Study Using Linked Primary and Secondary Care Databases. BMJ open 2021;11(5):e041566–e. doi: 10.1136/bmjopen-2020-041566 33952535 PMC8103369

[31] Fukami T, Koga H, Goto M, Ando M, Matsuoka S, Tohyama A, et al. Incidence and risk factors for postpartum hemorrhage among transvaginal deliveries at a tertiary perinatal medical facility in Japan. PLoS ONE [Electronic Resource]. 2019;14(1):e0208873. doi: 10.1371/journal.pone.0208873 30625154 PMC6326562

[32] Fullerton G, Danielian PJ, Bhattacharya S. Outcomes of pregnancy following postpartum haemorrhage. BJOG: an international journal of obstetrics and gynaecology. 2013;120(5):621–7. doi: 10.1111/1471-0528.12120 23339709

[33] Biguzzi E, Franchi F, Ambrogi F, Ibrahim B, Bucciarelli P, Acaia B, et al. Risk factors for postpartum hemorrhage in a cohort of 6011 Italian women. Thrombosis Research. 2012;129(4):e1–7. doi: 10.1016/j.thromres.2011.09.010 22018996

[34] Jardine J, Gurol-Urganci I, Harris T, Hawdon J, Pasupathy D, van der Meulen J, et al. Risk of postpartum haemorrhage is associated with ethnicity: A cohort study of 981 801 births in England. BJOG: an international journal of obstetrics and gynaecology. 2021. 10.1111/1471-0528.17051 34889021

[35] Rubio-Álvarez A, Molina-Alarcón M, Hernández-Martínez A. Incidence of postpartum anaemia and risk factors associated with vaginal birth. Women and birth: journal of the Australian College of Midwives. 2018;31(3):158–65. doi: 10.1016/j.wombi.2017.09.020 29107784

[36] van Stralen G, von Schmidt Auf Altenstadt JF, Bloemenkamp KW, van Roosmalen J, Hukkelhoven CW. Increasing Incidence of Postpartum Hemorrhage: The Dutch Piece of the Puzzle. Obstetric anesthesia digest. 2017;37(3):143–4. 10.1111/aogs.12950 27460955

[37] Bais JM, Eskes M, Pel M, Bonsel GJ, Bleker OP. Postpartum haemorrhage in nulliparous women: incidence and risk factors in low and high risk women. A Dutch population-based cohort study on standard (> or = 500 ml) and severe (> or = 1000 ml) postpartum haemorrhage. European Journal of Obstetrics, Gynecology, & Reproductive Biology. 2004;115(2):166–72. 15262350 10.1016/j.ejogrb.2003.12.008

[38] Buzaglo N, Harlev A, Sergienko R, Sheiner E. Risk factors for early postpartum hemorrhage (PPH) in the first vaginal delivery, and obstetrical outcomes in subsequent pregnancy. Journal of Maternal-Fetal & Neonatal Medicine. 2015;28(8):932–7. doi: 10.3109/14767058.2014.937698 25023434

[39] Chen BN, Wang D, Li JP, Zhang LY, Qiao C. Postpartum hemorrhage following cesarean delivery in women with a scarred uterus: A retrospective cohort study. Reproductive and Developmental Medicine. 2019;3(4):230–4.

[40] Eto H, Hasegawa A, Kataoka Y, Porter SE. Factors contributing to postpartum blood-loss in low-risk mothers through expectant management in Japanese birth centres. Women & Birth: Journal of the Australian College of Midwives. 2017;30(4):e158–e64. doi: 10.1016/j.wombi.2016.11.003 27876367

[41] Hiersch L, Bergel-Bson R, Asher D, Aviram A, Gabby-Benziv R, Yogev Y, et al. Risk factors for post-partum hemorrhage following vacuum assisted vaginal delivery. Archives of Gynecology & Obstetrics. 2017;295(1):75–80. doi: 10.1007/s00404-016-4208-5 27683268

[42] Shinar S, Schwartz A, Maslovitz S, Many A. How Long Is Safe? Setting the Cutoff for Uncomplicated Third Stage Length: A Retrospective Case-Control Study. Birth. 2016;43(1):36–41. doi: 10.1111/birt.12200 26555024

[43] Bateman BT, Berman MF, Riley LE, Leffert LR. The epidemiology of postpartum hemorrhage in a large, nationwide sample of deliveries. Anesthesia & Analgesia. 2010;110(5):1368–73. 20237047 10.1213/ANE.0b013e3181d74898

[44] Downe S, Finlayson K, Oladapo OT, Bonet M, Gülmezoglu AM. What matters to women during childbirth: A systematic qualitative review. PloS one. 2018;13(4):e0194906–e. doi: 10.1371/journal.pone.0194906 29664907 PMC5903648

[45] Meroz MR, Yu L-M, Sanders J, Rowe R. Preparedness for maternal and neonatal emergencies in UK midwifery units: A national survey using the UK Midwifery Study System (UKMidSS). Midwifery. 2022;110:103336-. doi: 10.1016/j.midw.2022.103336 35439651

[46] Mavrides E, Allard S, Chandraharan E, Collins P, Green L, Hunt B, et al. Prevention and Management of Postpartum Haemorrhage: Green-top Guideline No. 52. BJOG: an international journal of obstetrics and gynaecology. 2017;124(5):e106–e49. doi: 10.1111/1471-0528.14178 27981719

