# Supplementary material for: Postpartum haemorrhage occurring in UK midwifery units: A national population-based case-control study to investigate incidence, risk factors and outcomes
Source: PLoS One. 2023 Oct 5;18(10):e0291795. doi: 10.1371/journal.pone.0291795 (PMC10553245; doi:10.1371/journal.pone.0291795)
Supplement: S1 Table — (DOCX) [file pone.0291795.s001.docx]

**Table S1. Maternal sociodemographic characteristics among women who had a PPH requiring transfer, according to whether they received ‘enhanced treatment or care’**

|  | **No ‘enhanced treatment or care’ (n =1,131)** | | **‘Enhanced treatment or care’**  **(n = 370)** | | **Unadjusted ORs** | | **p value** |
| --- | --- | --- | --- | --- | --- | --- | --- |
|  | **n** | **%** | **n** | **%** | **OR** | **95% CI** |  |
| **Maternal age** | | | | | | | 0.364 |
| <20 | 32 | 2.8 | 16 | 4.3 | 1.82 | (0.97-3.44) |  |
| 20-24 | 150 | 13.3 | 55 | 14.9 | 1.34 | (0.85-2.09) |  |
| 25-29 | 328 | 29.0 | 90 | 24.3 | 1 | . |  |
| 30-34 | 417 | 37.1 | 135 | 36.5 | 1.18 | (0.89-1.56) |  |
| 35-40 | 184 | 16.2 | 69 | 18.7 | 1.37 | (0.93-2.02) |  |
| >40 | 20 | 1.8 | 5 | 1.4 | 0.91 | (0.30-2.73) |  |
| Missing | 0 | . | 0 |  | . | . |  |
| **Smoking status** | | | | | | | 0.234 |
| Did not smoke during pregnancy | 1024 | 90.5 | 327 | 88.4 | 1 | . |  |
| Smoked during pregnancy | 88 | 7.8 | 33 | 8.9 | 1.17 | (0.72-1.89) |  |
| Missing | 19 | 1.7 | 10 | 2.7 | 1.64 | (0.90-3.02) |  |
| **Area deprivation quintile** | | | | | | | 0.473 |
| 1st (least deprived) | 285 | 25.4 | 95 | 26.0 | 1 | . |  |
| 2^nd^ | 217 | 19.3 | 84 | 23.0 | 1.16 | (0.84-1.61) |  |
| 3^rd^ | 213 | 19.0 | 57 | 15.6 | 0.80 | (0.54-1.17) |  |
| 4^th^ | 209 | 18.6 | 65 | 17.8 | 0.93 | (0.64-1.35) |  |
| 5^th^ (most deprived) | 200 | 17.8 | 65 | 17.8 | 0.98 | (0.69-1.37) |  |
| Missing | 7 | . | 4 | . | . | . |  |
| **Ethnicity** | | | | | | | 0.217 |
| White (UK and Ireland) | 768 | 67.9 | 229 | 61.9 | 1 | . |  |
| White (other) | 143 | 12.7 | 46 | 12.4 | 1.01 | (0.68-1.72) |  |
| Asian | 122 | 10.8 | 49 | 13.2 | 1.34 | (0.89-2.03) |  |
| Black | 43 | 3.8 | 21 | 5.7 | 1.64 | (0.96-2.78) |  |
| Other | 55 | 4.9 | 25 | 6.8 | 1.52 | (0.94-2.47) |  |
| Missing | 0 | . | 0 | . | . | . |  |
| **Socioeconomic status** | | | | | | | 0.264 |
| Higher managerial | 387 | 34.2 | 139 | 37.6 | 1 | . |  |
| Intermediate | 201 | 17.8 | 63 | 17.0 | 0.87 | (0.65-1.18) |  |
| Routine and manual | 241 | 21.3 | 94 | 25.4 | 1.09 | (0.76-1.56) |  |
| Employed, occupation unknown | 81 | 7.2 | 22 | 6.0 | 0.76 | (0.45-1.27) |  |
| Unemployed/ student | 64 | 5.7 | 16 | 4.3 | 0.70 | (0.39-1.26) |  |
| Not recorded | 157 | 13.9 | 36 | 9.7 | 0.64 | (0.40-1.01) |  |
