# Supplementary material for: Postpartum haemorrhage occurring in UK midwifery units: A national population-based case-control study to investigate incidence, risk factors and outcomes
Source: PLoS One. 2023 Oct 5;18(10):e0291795. doi: 10.1371/journal.pone.0291795 (PMC10553245; doi:10.1371/journal.pone.0291795)
Supplement: S2 Table — (DOCX) [file pone.0291795.s002.docx]

**Table S2. Pre-existing clinical characteristics among women who had a PPH requiring transfer, according to whether they received ‘enhanced treatment or care’**

|  | **No ‘enhanced treatment or care’**  **(n =1,131)** | | **‘Enhanced treatment or care’**  **(n =370)** | | **Unadjusted ORs** | | **p value** |
| --- | --- | --- | --- | --- | --- | --- | --- |
|  | **n** | **%** | **n** | **%** | **OR** | **95% CI** |  |
| **BMI at booking** | | | | | | | 0.052 |
| < 18.5 | 26 | 2.3 | 16 | 4.3 | 2.07 | (1.15-3.70) |  |
| 18.5-24.9 | 608 | 53.8 | 181 | 48.9 | 1 | . |  |
| 25-29.9 | 326 | 28.8 | 106 | 28.7 | 1.09 | (0.84-1.42) |  |
| 30-35 | 120 | 10.6 | 48 | 13.0 | 1.34 | (0.96-1.89) |  |
| >35 | 24 | 2.1 | 6 | 1.6 | 0.83 | (0.33-2.09) |  |
| BMI not recorded | 27 | 2.4 | 13 | 3.5 | 1.62 | (0.83-3.17) |  |
| **Parityº** | | | | | | | 0.726 |
| 0 | 559 | 49.4 | 191 | 51.6 | 1.10 | (0.87-1.39) |  |
| 1 | 418 | 37.0 | 130 | 35.1 | 1 | . |  |
| 2 or more | 154 | 13.6 | 49 | 13.2 | 1.02 | (0.71-1.48) |  |
| Missing | 0 | . | 0 | . | . | . |  |
| **Pre-existing medical risk factors*** | | | | | | | 0.431 |
| None | 1,109 | 98.1 | 365 | 98.7 | 1 | . |  |
| One or more | 22 | 2.0 | 5 | 1.4 | 0.69 | (0.27-1.73) |  |
| **Problems in a previous pregnancy**† | | | | | | | 0.976 |
| No previous complication | 495 | 86.5 | 155 | 87.6 | 1 | . |  |
| Previous PPH | 56 | 9.8 | 18 | 10.1 | 1.03 | (0.58-1.80) |  |
| Previous complication other than PPH | 21 | 3.7 | 6 | 3.4 | 0.91 | (0.34-2.43) |  |

º Parity refers to the number of previous pregnancies carried to at least 24 completed weeks’ gestation

* Pre-existing medical risk factors comprises: Hypertension, Confirmed cardiac disease, Thromboembolic disorder, atypical antibodies, hyperthyroidism, diabetes, renal disease and epilepsy. Includes multiparous women only.

† Problems in a previous pregnancy comprises: Retained placenta requiring manual removal, Caesarean section and uterine surgery excluding Caesarean section. Includes multiparous women only.
