# Supplementary material for: Postpartum haemorrhage occurring in UK midwifery units: A national population-based case-control study to investigate incidence, risk factors and outcomes
Source: PLoS One. 2023 Oct 5;18(10):e0291795. doi: 10.1371/journal.pone.0291795 (PMC10553245; doi:10.1371/journal.pone.0291795)
Supplement: S3 Table — (DOCX) [file pone.0291795.s003.docx]

**Table S3. Clinical characteristics arising during pregnancy among women who had a PPH requiring transfer, according to whether they received ‘enhanced treatment or care’**

|  | **No ‘enhanced treatment or care’ (n =1,131)** | | **‘Enhanced treatment or care’**  **(n = 370)** | | **Unadjusted ORs** | | **p value** |
| --- | --- | --- | --- | --- | --- | --- | --- |
|  | **n** | **%** | **n** | **%** | **OR** | **95% CI** |  |
| **Current pregnancy problem*** | | | | | | | 0.477 |
| None | 1,054 | 93.2 | 349 | 94.3 | 1 | . |  |
| One or more | 77 | 6.8 | 21 | 5.7 | 0.81 | (0.48-1.40) |  |
| **Sex of baby** | | | | | | | 0.600 |
| Male | 544 | 48.2 | 173 | 46.8 | 1.06 | (0.85-1.32) |  |
| Female | 584 | 51.8 | 197 | 53.2 | 1 | . |  |
| Missing | 3 | . | 0 | . | . | . |  |
| **Gestational age (weeks)** | | | | | | | 0.429 |
| 36-37 | 28 | 2.5 | 10 | 2.7 | 1.11 | (0.52-2.40) |  |
| 38 | 111 | 9.8 | 32 | 8.7 | 0.90 | (0.60-1.34) |  |
| 39 | 277 | 24.5 | 81 | 21.9 | 0.91 | (0.70-1.19) |  |
| 40 | 446 | 39.4 | 143 | 38.7 | 1 | . |  |
| 41+ | 269 | 23.8 | 104 | 28.1 | 1.21 | (0.91-1.60) |  |
| Missing | 0 | . | 0 | . | . | . |  |

* Current pregnancy problem: BMI at booking >35, post-term (>42 weeks) Anaemia, Group B Streptococcus, Antepartum haemorrhage, pre-eclampsia/pregnancy-induced hypertension, gestational diabetes, and malpresentation (breech or transverse lie).
