# Supplementary material for: Postpartum haemorrhage occurring in UK midwifery units: A national population-based case-control study to investigate incidence, risk factors and outcomes
Source: PLoS One. 2023 Oct 5;18(10):e0291795. doi: 10.1371/journal.pone.0291795 (PMC10553245; doi:10.1371/journal.pone.0291795)
Supplement: S4 Table — (DOCX) [file pone.0291795.s004.docx]

**Table S4. Intrapartum factors among women who had a PPH requiring transfer, according to whether they received ‘enhanced treatment or care’**

|  | **No ‘enhanced treatment or care’ (n = 1,131)** | | **‘Enhanced treatment or care’ (n = 370)** | | **Unadjusted ORs** | |  |
| --- | --- | --- | --- | --- | --- | --- | --- |
|  | **n** | **%** | **n** | **%** | **OR** | **95% CI** | **p value** |
| **Stage of labour at start of care** | | | | | | | 0.181 |
| Latent stage | 193 | 17.1 | 77 | 20.9 | 1.22 | (0.92-1.63) |  |
| Active 1st stage | 809 | 71.8 | 263 | 71.3 | 1 | . |  |
| Passive 2nd stage | 37 | 3.3 | 9 | 2.4 | 0.75 | (0.36-1.56) |  |
| Active 2nd stage | 88 | 7.8 | 20 | 5.4 | 0.69 | (0.40-1.22) |  |
| Missing | 4 | . | 1 | . | . | . |  |
| **Induction of labour** |  | | | | | | 0.945 |
| No | 1,062 | 94.2 | 348 | 94.3 | 1 | . |  |
| Yes | 63 | 5.6 | 21 | 5.6 | 1.02 | (0.63-1.65) |  |
| Missing | 6 | . | 1 | . | . | . |  |
| **Maternal complications identified at the start of labour care*** | | | | | | | 0.109 |
| None | 1,195 | 97.3 | 362 | 98.1 | 1 | . |  |
| One or more | 31 | 2.8 | 7 | 1.9 | 0.68 | (0.30-1.54) |  |
| Missing | 5 | . | 1 | . | . | . |  |
| **Fetal complications identified at the start of labour care†** | | | | | | | 0.749 |
| None | 1088 | 96.6 | 358 | 97.0 | 1 | . |  |
| One or more | 38 | 3.4 | 11 | 3.0 | 0.88 | (0.40-1.93) |  |
| Missing | 5 | . | 1 | . | . | . |  |
| **Maternal complications identified during labour (before birth)º** | | | | | | | 0.357 |
| None | 1,103 | 97.5 | 355 | 96.0 | 1 | . |  |
| One or more | 28 | 2.5 | 15 | 4.1 | 1.66 | (0.89-3.10) |  |
| Missing | 0 | . | 0 | . | . | . |  |
| **Fetal complications identified during labour (before birth)^¶^** | | | | | | | 0.235 |
| None | 1019 | 90.1 | 341 | 92.1 | 1 | . |  |
| One or more | 112 | 9.9 | 29 | 7.8 | 0.77 | (0.51-1.18) |  |
| Missing | 0 | . | 0 | . | . | . |  |
