# Supplementary material for: Postpartum haemorrhage occurring in UK midwifery units: A national population-based case-control study to investigate incidence, risk factors and outcomes
Source: PLoS One. 2023 Oct 5;18(10):e0291795. doi: 10.1371/journal.pone.0291795 (PMC10553245; doi:10.1371/journal.pone.0291795)
Supplement: S5 Table — (DOCX) [file pone.0291795.s005.docx]

**Table S5. Birth-related factors among women who had a PPH requiring transfer, according to whether they received ‘enhanced treatment or care’**

|  | **No ‘enhanced treatment or care’ (n = 1,131)** | | **‘Enhanced treatment or care’ (n = 370)** | | **Unadjusted ORs** | | **p value** |
| --- | --- | --- | --- | --- | --- | --- | --- |
|  | **n** | **%** | **n** | **%** | **OR** | **95% CI** |  |
| **Labour/birth in water** | | | | | | | 0.113 |
| No immersion in water | 307 | 27.2 | 118 | 32.0 | 1 | . |  |
| Immersion in water for labour, land birth | 192 | 17.0 | 71 | 19.2 | 0.96 | (0.65-1.41) |  |
| Birth in water | 628 | 55.7 | 180 | 48.8 | 0.75 | (0.55-1.01) |  |
| Missing | 4 | . | 1 | . |  |  |  |
| **Birth mode** | | | | | | | 0.480 |
| Spontaneous vertex birth | 1,098 | 97.8 | 358 | 97.3 | 1 | . |  |
| Instrumental | 25 | 2.2 | 10 | 2.7 | 1.22 | (0.69-2.16) |  |
| Missing | 8 | . | 2 | . | . | . |  |
| **Duration of third stage of labour** | | | | | | | <0.001 |
| < 60 minutes | 956 | 84.5 | 284 | 76.8 | 1 | . |  |
| ≥60 minutes | 148 | 13.1 | 79 | 21.4 | 1.14 | (1.36-2.45) |  |
| Missing | 27 | 2.4 | 7 | 1.9 | 2.05 | (0.52-2.52) |  |
| **Perineal tear** | | | | | | | 0.702 |
| <3^rd^ degree tear | 969 | 86.0 | 321 | 86.8 | 1 | . |  |
| 3^rd^ or 4^th^ degree tear | 158 | 14.0 | 49 | 13.2 | 0.89 | (0.67-1.31) |  |
| Missing | 4 | . | 0 | . | . | . |  |
| **Syntocinon/ Syntometrine for 3rd stage management** | | | | | | | 0.590 |
| Yes | 976 | 13.5 | 315 | 14.6 | 1 | . |  |
| No | 151 | 86.6 | 54 | 85.4 | 0.90 | (0.62-1.31) |  |
| Missing | 4 | . | 1 | . | . | . |  |
| **Birthweight (g)** | | | | | | | 0.246 |
| <3000 | 88 | 7.8 | 24 | 6.5 | 0.92 | (0.58-1.46) |  |
| 3000-3499 | 392 | 34.8 | 116 | 31.4 | 1 | . |  |
| 3500-3999 | 468 | 41.5 | 160 | 43.2 | 1.15 | (0.86-1.56) |  |
| ≥4000 | 180 | 16.0 | 70 | 18.9 | 1.31 | (0.96-1.80) |  |
| Missing | 3 | . | 0 | . | . | . |  |
