# Supplementary material for: Postpartum haemorrhage occurring in UK midwifery units: A national population-based case-control study to investigate incidence, risk factors and outcomes
Source: PLoS One. 2023 Oct 5;18(10):e0291795. doi: 10.1371/journal.pone.0291795 (PMC10553245; doi:10.1371/journal.pone.0291795)
Supplement: S6 Table — (DOCX) [file pone.0291795.s006.docx]

Table S6. Neonatal outcomes among cases and controls

|  | **Controls** | | **Cases** | |
| --- | --- | --- | --- | --- |
|  | **n** | **%** | **n** | **%** |
| Apgar score <7, at 5 minutes | 12 | 0.8 | 20 | 1.3 |
| Admission to higher level of care | 25 | 1.5 | 41 | 2.7 |
| Neonatal morbidity reported* | 9 | 0.6 | 31 | 2.0 |

* Neonatal morbidity includes: birth injury, congenital anomaly, jaundice, sepsis/infection, meconium at birth, respiratory distress
