# Supplementary material for: Postpartum haemorrhage occurring in UK midwifery units: A national population-based case-control study to investigate incidence, risk factors and outcomes
Source: PLoS One. 2023 Oct 5;18(10):e0291795. doi: 10.1371/journal.pone.0291795 (PMC10553245; doi:10.1371/journal.pone.0291795)
Supplement: S7 Table — (DOCX) [file pone.0291795.s007.docx]

Table S7. Risk factors for PPH requiring transfer to obstetric care among cases according to the type on unit in which they gave birth

|  | **FMU**  **n = 99** | | **AMU**  **n = 1,402** | | **p value** |
| --- | --- | --- | --- | --- | --- |
|  | **n** | **%** | **n** | **%** |  |
| **Smoking status** |  |  |  |  | <0.001 |
| Did not smoke during pregnancy | 87 | 87.9 | 1264 | 90.2 |  |
| Smoked during pregnancy | 5 | 5.0 | 116 | 8.3 |  |
| Missing | 7 | 7.1 | 22 | 1.6 |  |
| **Parity** |  |  |  |  | 0.640 |
| 0 | 54 | 54.6 | 696 | 49.6 |  |
| 1 | 33 | 33.3 | 515 | 36.7 |  |
| 2+ | 12 | 12.1 | 191 | 13.6 |  |
| Missing | 0 | . | 0 | . |  |
| **Previous pregnancy complication** |  |  |  |  | 0.214 |
| No previous complication | 41 | 91.1 | 609 | 86.3 |  |
| Previous PPH | 2 | 4.4 | 72 | 10.2 |  |
| Previous complication other than PPH | 2 | 4.4 | 25 | 3.5 |  |
| **Gestational age** |  |  |  |  | 0.531 |
| 36-37 | 4 | 4.0 | 34 | 2.4 |  |
| 38 | 12 | 12.1 | 131 | 9.3 |  |
| 39 | 27 | 27.3 | 331 | 23.6 |  |
| 40 | 34 | 34.3 | 555 | 39.6 |  |
| 41+ | 22 | 22.2 | 351 | 25.0 |  |
| Missing | 0 | . | 0 | . |  |
| **Birth mode** |  |  |  |  | 0.110 |
| Spontaneous vertex or vaginal breech birth | 99 | 100.0 | 1357 | 97.5 |  |
| Instrumental birth | 0 | 0 | 35 | 2.5 |  |
| Missing | 0 | . | 10 | . |  |
| **Duration of third stage of labour** |  |  |  |  | <0.001 |
| < 60 minutes | 77 | 77.8 | 1163 | 82.9 |  |
| ≥ 60 minutes | 12 | 12.1 | 215 | 15.3 |  |
| Missing | 10 | 10.1 | 24 | 1.7 |  |
| **Syntocinon/ Syntometrine for 3rd stage management** |  |  |  |  | 0.462 |
| Yes | 16 | 16.2 | 189 | 13.5 |  |
| No | 83 | 83.8 | 1208 | 86.5 |  |
| Missing | 0 | . | 5 | . |  |
| **Perineal tear** |  |  |  |  | 0.319 |
| <3rd degree tear or no tear | 82 | 82.8 | 1208 | 86.4 |  |
| 3rd or 4th degree tear | 17 | 17.2 | 190 | 13.6 |  |
| Missing | 0 | . | 4 | . |  |
| **Birthweight (gm)** |  |  |  |  | 0.417 |
| <3000 | 6 | 6.1 | 106 | 7.6 |  |
| 3000-3499 | 27 | 27.3 | 481 | 34.3 |  |
| 3500-3999 | 47 | 47.5 | 581 | 41.5 |  |
| ≥4000 | 19 | 19.2 | 231 | 16.5 |  |
| Missing | 0 | . | 3 | . |  |

* Among multiparous women only
