# Supplementary material for: Postpartum haemorrhage occurring in UK midwifery units: A national population-based case-control study to investigate incidence, risk factors and outcomes
Source: PLoS One. 2023 Oct 5;18(10):e0291795. doi: 10.1371/journal.pone.0291795 (PMC10553245; doi:10.1371/journal.pone.0291795)
Supplement: S8 Table — (DOCX) [file pone.0291795.s008.docx]

Table S8. Risk factors for PPH requiring transfer to obstetric care among controls according to the type on unit in which they gave birth

|  | **FMU**  **n = 94** | | **AMU**  **n = 1381** | | **p value** |
| --- | --- | --- | --- | --- | --- |
|  | **n** | **%** | **n** | **%** |  |
| **Smoking status** |  |  |  |  | 0.39 |
| Did not smoke during pregnancy | 77 | 81.9 | 1197 | 86.7 |  |
| Smoked during pregnancy | 15 | 16.0 | 156 | 11.3 |  |
| Missing | 2 | 12.1 | 28 | 2.0 |  |
| **Parity** |  |  |  |  | 0.262 |
| 0 | 40 | 42.6 | 473 | 34.3 |  |
| 1 | 37 | 39.4 | 617 | 44.7 |  |
| 2+ | 17 | 18.1 | 291 | 21.1 |  |
| Missing | 0 | . | 0 | . |  |
| **Previous pregnancy complication*** |  |  |  |  | 0.421 |
| No previous complication | 53 | 98.1 | 856 | 96.0 |  |
| Previous PPH | 1 | 1.9 | 36 | 4.0 |  |
| Previous complications other then PPH | 0 | 0 | 16 | 1.8 |  |
| **Gestational age** |  |  |  |  | 0.728 |
| 36-37 | 3 | 3.2 | 63 | 4.6 |  |
| 38 | 14 | 14.9 | 153 | 11.1 |  |
| 39 | 22 | 23.4 | 377 | 27.3 |  |
| 40 | 39 | 41.5 | 552 | 40.0 |  |
| 41-43 | 16 | 17.0 | 235 | 17.0 |  |
| Missing | 0 | . | 1 | . |  |
| **Birth mode** |  |  |  |  | 0.410 |
| Spontaneous vertex or vaginal breech birth | 93 | 100.0 | 1368 | 99.3 |  |
| Instrumental birth | 0 | 0 | 10 | 0.7 |  |
| Missing | 1 | . | 3 | . |  |
| **Duration of third stage of labour** |  |  |  |  | <0.001 |
| < 60 minutes | 79 | 84.0 | 1320 | 95.6 |  |
| ≥ 60 minutes | 10 | 10.6 | 43 | 3.1 |  |
| Missing | 5 | 5.3 | 18 | 1.3 |  |
| **Syntocinon/ Syntometrine for 3rd stage management** |  |  |  |  | 0.010 |
| Yes | 66 | 71.0 | 1128 | 81.8 |  |
| No | 27 | 29.0 | 251 | 18.2 |  |
| Missing | 1 | . | 2 | . |  |
| **Perineal tear** |  |  |  |  | 0.600 |
| <3rd degree tear or no tear | 91 | 97.9 | 1336 | 96.9 |  |
| 3rd or 4th degree tear | 2 | 2.1 | 43 | 3.1 |  |
| Missing | 1 | . | 2 | . |  |
| **Birthweight (gm)** |  |  |  |  | 0.061 |
| <3000 | 20 | 21.5 | 172 | 12.5 |  |
| 3000-3499 | 34 | 36.6 | 610 | 44.3 |  |
| 3500-3999 | 32 | 34.4 | 453 | 32.9 |  |
| ≥4000 | 7 | 7.5 | 143 | 10.4 |  |
| Missing | 1 | . | 3 | . |  |

* Among multiparous women only
