# Supplementary material for: Postpartum haemorrhage occurring in UK midwifery units: A national population-based case-control study to investigate incidence, risk factors and outcomes
Source: PLoS One. 2023 Oct 5;18(10):e0291795. doi: 10.1371/journal.pone.0291795 (PMC10553245; doi:10.1371/journal.pone.0291795)
Supplement: S9 Table — (DOCX) [file pone.0291795.s009.docx]

Table S9. Blood loss among cases and controls by unit type

|  | **FMU**  **n = 193** | | **AMU**  **n = 2,783** | | **p value** |
| --- | --- | --- | --- | --- | --- |
|  | **n** | **%** | **n** | **%** |  |
| **Blood loss among controls (mL)** |  |  |  |  | 0.627 |
| <500 | 88 | 96.7 | 1289 | 93.5 |  |
| 500 | 1 | 1.1 | 45 | 3.3 |  |
| 501-999 | 2 | 2.2 | 41 | 3.0 |  |
| 1000-1499 | 0 | 0.0 | 3 | 0.2 |  |
| Missing | 3 | . | 3 | . |  |
| **Blood loss among cases (mL)** |  |  |  |  | 0.140 |
| <500 | 1 | 1.1 | 1 | 0.1 |  |
| 500 | 3 | 3.0 | 58 | 4.2 |  |
| 501-999 | 38 | 39.4 | 516 | 36.9 |  |
| 1000-1499 | 31 | 31.3 | 486 | 34.8 |  |
| 1500 | 26 | 26.3 | 337 | 24.1 |  |
| Missing | 0 | . | 4 | . |  |
