# Supplementary material for: Postpartum haemorrhage occurring in UK midwifery units: A national population-based case-control study to investigate incidence, risk factors and outcomes
Source: PLoS One. 2023 Oct 5;18(10):e0291795. doi: 10.1371/journal.pone.0291795 (PMC10553245; doi:10.1371/journal.pone.0291795)
Supplement: Attachment — Submitted filename: Response to Reviewers.docx [file pone.0291795.s010.docx]

**PLOS ONE revision comments and responses**

| **Comment** | **Response** |
| --- | --- |
| 1. Please ensure that your manuscript meets PLOS ONE's style requirements, including those for file naming. The PLOS ONE style templates can be found at  <https://journals.plos.org/plosone/s/file?id=wjVg/PLOSOne_formatting_sample_main_body.pdf> and <https://journals.plos.org/plosone/s/file?id=ba62/PLOSOne_formatting_sample_title_authors_affiliations.pdf> | We have checked the manuscript against the journal’s style requirements. File names for Figures 1 and 2 have been revised and re-uploaded. |
| 2. Please amend your current ethics statement to address the following concerns:  a) Did participants provide their written or verbal informed consent to participate in this study?  b) If consent was verbal, please explain i) why written consent was not obtained, ii) how you documented participant consent, and iii) whether the ethics committees/IRB approved this consent procedure. | The ethics statement has been amended to include a sentence reflecting the fact that this study used anonymised data collected directly from medical records in participating units, so consent was not required. |
| 3. We note that the grant information you provided in the ‘Funding Information’ and ‘Financial Disclosure’ sections do not match.  When you resubmit, please ensure that you provide the correct grant numbers for the awards you received for your study in the ‘Funding Information’ section | We have completed the ‘Financial Disclosure’ statement in full and the correct grant numbers for the awards received, where relevant, were provided in the original submission. There is no grant number associated with the University of Oxford Nuffield Department of Population Health DPhil Scholarship.  We have been unable to find a separate ‘Funding Information’ section in the submission system.  Please advise if any further information is still required. |
| 4. Please include your full ethics statement in the ‘Methods’ section of your manuscript file. In your statement, please include the full name of the IRB or ethics committee who approved or waived your study, as well as whether or not you obtained informed written or verbal consent. If consent was waived for your study, please include this information in your statement as well. | The full name of the ethics committee that approved the study was included in the original manuscript.  We have added a sentence about consent to the ethics statement. |
| 5. One of the noted authors is a group or consortium “UKMidSS Steering Group”. In addition to naming the author group, please list the individual authors and affiliations within this group in the acknowledgments section of your manuscript. Please also indicate clearly a lead author for this group along with a contact email address. | The individual authors in the UKMidSS Steering Group and their affiliations were provided in the acknowledgements section of the original transcript.  The lead author for this group is Rachel Rowe and we have now indicated this in the title page and provided a contact email. |
| 6. Please review your reference list to ensure that it is complete and correct. If you have cited papers that have been retracted, please include the rationale for doing so in the manuscript text, or remove these references and replace them with relevant current references. Any changes to the reference list should be mentioned in the rebuttal letter that accompanies your revised manuscript. If you need to cite a retracted article, indicate the article’s retracted status in the References list and also include a citation and full reference for the retraction notice. | The reference list has been checked and minor changes made where required. We have not identified any retracted papers cited in this work. |
| **Reviewer 1** | |
| This is a prospective national (England UK) population-based case-control study in all MUs in the UK using 27 the UK Midwifery Study System (UKMidSS). This has become a highly successful method of capturing prospective data from maternity settings in the UK and has informed practice relating to many different obstetric complications. There is a good series of highly referenced papers that have previously published using this methodology with high impact. Previously published PPH papers from UKOSS have been very informative and this paper would add to this series of publications. | Thank you for your positive comment. |
| The study was supposed to run over 12 months based on the incidence of PPH transfers from midwifery to obstetric settings from the literature but was stopped at 6 months because of the COVID pandemic. This may have led to some reduction of impact relating to some of the outcomes/recommendations but as the incidence of transfer was higher than previously reported the study seemed adequately powered. | We agree that the early termination of data collection may have reduced the impact of some findings but that overall the study was still adequately powered to investigate the main research questions. |
| Overall, I found the writing style quite long with the frequent use of the pronoun “we”. I feel that the paper would benefit from a more focused writing style. Consideration should be made to removing “we.... did this” to “...was undertaken” or something similar | We have reviewed the paper carefully and revised it in several places to reduce the number of times the word “we” is used. |
| As journals are international I think there needs to be a clear but concise explanation of how and why the UK maternity system is set up with MLC/CLU. | We are mindful of the need to appropriately describe the way that maternity care is organised in the UK for international readers. We have revised the first paragraph where we had described the different settings where women can give birth, adding a short explanation of the policy context and the justification for the current policy related to choice of place of birth. |
| It is also somewhat confusing in the text as clearly some high-risk women were admitted to the MLU which is clearly contrary to national guidance but can be the result of local practice or personal preference. Again, this requires brief explanation. | A brief explanation of the differences in admission criteria between MUs and the implications this may have for the risk profile of women who give birth in MUs has been added to the introduction, from line 70 of the manuscript. |
| In the discussion there needs to be clearly written highlights to what this paper adds to the literature. I think there are many. | Thank you for this positive feedback. We have re-phrased some of the introduction of the discussion to highlight what this paper adds to the literature. |
| I would also like to see more of an acknowledgement that a PPH rate of 3% in a low-risk population is significant and not without maternal morbidity. One lady had a 5L PPH. In addition, the rate appears to be higher than previously reported. Part of the conclusion must therefore to maintain safe care there should be robust guidelines on MLU management of PPH and robust rapid transfer policies. I found the tone too reassuring. | We agree that 3.7% is not negligible in this population and has potential consequences for maternal morbidity. While we have discussed the higher than expected incidence, and potential reasons for this, we agree that the potential implications for MLU management and guidelines did not come out clearly enough in our discussion. We have now added to the discussion and conclusions to emphasise this point more clearly. |
| **Reviewer 2** | |
| This paper presents a contemporaneous snapshot of transfers form MUs following or for treatment for PPH. As such this is novel as these data have not previously been reported (as identified BirthPlace reported all transfers). The high response rate from units was impressive and clearly identities the effectiveness of the UKMidSS methodology. | Thank you for your positive comment. We agree that the high response rate is a strength of this study and acknowledge the important contribution of reporting midwives in this research. |
| Selection of the preceding birth as a control, with no adjustment, for example, for parity was interesting, and could have been better rationalised - ie, by definition these are all 'low risk' women, and may have contributed to the imbalance of primiparous women included in the control group. Especially given the general advice that MU care is associated with fewer interventions and better outcomes for multiparous women. | We are not sure if we fully understand this comment. Our design used what is sometimes described as ‘cosmetic matching’ whereby the control was selected as the preceding birth for each case (see Bland, J M, & Altman, D G. (1994). Statistics notes: Matching. BMJ, 309(6962), 1128). This approach results in a control group that is broadly representative of the population of women who give birth in MUs in the UK, on the basis that time of birth is not likely to be associated with the outcome of interest (PPH). By selecting the birth preceding the case we also ensured that care and management of the control could not have been affected by the occurrence of the case.  The proportion of multiparous women in the control group is in line with expected proportions based on other studies (See Rowe et al 2021 and Rowe et al 2018, in the reference list)  In terms of adjustment for parity, this occurred at the multivariable level. |
| The "risk profile' of this cohort is interesting and some rationale or explanation around this might have proved useful. For example many AMUs and FMUs have guidelines excluding women with high BMIs, or pre-labour complications and yet there were several included in this cohort. | The risk profile of the study cohort reflects the risk profile of women who give birth in MUs in the UK, as explained above.  We have added a brief section to the introduction that addresses variation between national guidance and typical admissions criteria in MUs in practice, resulting in a small proportion of women with medical risk factors or complications in the cohort (lines 68 -74). |
| Table 2 I calculate the incidence related to previous PPH in cases as 9.8% not 10.2 as reported. This is still strongly significant but changes the OR slightly (2.74 95%CI 1.81 to 4.10). I note the numbers reported in Table 4 as adjusted OR are more similar. | The slight difference between what the reviewer calculated and what is reported is because the denominator for this variable excluded women who had a previous pregnancy complication other than PPH. On reflection we recognise that this is potentially confusing, so we have revised our approach and combined these variables into one, making the comparison group clearer, and have updated the tables to reflect this. |
| Table 3: Indicates 84 cases and 56 controls had labour induced. Is there any explanation around this? Given that induction of labour is a medical procedure and initiation is outside the midwife's scope of practice. | Data were not available about the indication or method of induction. Previous MU research indicates that birth in an MU after induction does occur in a minority of cases (Rowe et al 2018; Rowe et al 2021). |
| Similarly there were (a few) women (total n= 54) with complications identified before labour, more than twice as many of these were in the cases rather than the controls. Similarly there were 77 incidences where fetal complications were identified before labor, and again more of these were in the case group than the control (1.9% versus 3.2%). Similar disparities were seen in complications identified during labour but there are no references to these in the discussion. | As the reviewer notes, these factors were associated with PPH at the univariable level. However, these associations did not remain after adjustment for other factors. We have added a short section in the discussion that addresses these intrapartum factors. |
| The authors reported water immersion in labour was a significant risk factor for PPH, but this is not expanded in the discussion. Given that many women choose birthing in MUs in order to use water immersion for labour and/or birth, this could be an important message about risk factors. | We agree that women’s choices may be informed by the evidence presented in this paper. We are also mindful however that the discussion section is already quite long and we do not want to put undue emphasis on factors that were not significantly associated with PPH after adjustment for other factors. |
| The incidence of instrumental vaginal births in these low risk settings is explained in the discussion.  The incidence of 3rd and 4th degree tears appears high, and although contributory to transfer for PPH, these women are more likely transferred for repair. | We don’t have data from a similar population against which to compare the incidence of 3^rd^ or 4^th^ degree tear. Because our case definition included women for whom either the primary or secondary reason for transfer was PPH, women who were transferred for repair of a 3^rd^ of 4^th^ degree tear would have been included as cases if the secondary reason for transfer was PPH. |
| Was the higher incidence at later gestation due to these women being induced? As this is a MU study one would assume all women were managed expectantly but the number of IOLs suggests otherwise, and were some additional women augmented, which has not been documented? Given the known associations between syntocinon use in the 1st and 2nd stage of labour and subsequent PPH, this would not be surprising. | Induction of labour was not significant at the multivariable level and there is no evidence in our data that the increased risk of PPH among women giving birth at later gestational age was associated with induction. As this study only includes women who gave birth in a midwifery unit, where augmentation of labour is not carried out, none of these women would have had their labour augmented. |
| It is a shame this study was stopped prematurely, as I am sure some of these issues are down to smaller numbers than anticipated, although almost 40000 women is not a small number! | We agree that some additional analyses may have been possible had the study been able to include a full year of data, however, as noted by the reviewer, the study was still a substantial size and adequately powered to investigate the research questions. |
| DISCUSSION: This appears to omit any comments about the profile of women birthing in MUs. There are several in this cohort that would appear to be higher risk than low risk and therefore some discussion around choice and availability should be included. Note that the authors do state that transfer was influenced by staffing levels.  I think there are also considerations around workforce skills and the impact that plays in transfer, and care provided and these are not alluded to in the discussion. | We have now added some discussion about the risk profile of women who give birth in a midwifery unit, in line with addressing the similar comments from Reviewer 1.  We did not collect data about the skill levels or staffing levels of midwifery units during the study and are therefore reluctant to speculate further on any potential associations with transfers for PPH. |
