# Supplementary material for: Postpartum haemorrhage occurring in UK midwifery units: A national population-based case-control study to investigate incidence, risk factors and outcomes
Source: PLoS One. 2023 Oct 5;18(10):e0291795. doi: 10.1371/journal.pone.0291795 (PMC10553245; doi:10.1371/journal.pone.0291795)
Supplement: Attachment — Submitted filename: Review Comments to Authors R1.docx [file pone.0291795.s011.docx]

**Comments to the Author**

The authors investigate the incidence, risk factors and outcomes of national population-based case-control in UK midwifery units. As per the revised contributions and the present form of manuscript is recommended.
